# 3D reconstruction of murine mitochondria reveals changes in structure during aging linked to the MICOS complex

**DOI:** 10.1111/acel.14009

**Published:** 2023-11-13

**Authors:** Zer Vue, Edgar Garza‐Lopez, Kit Neikirk, Prasanna Katti, Larry Vang, Heather Beasley, Jianqiang Shao, Andrea G. Marshall, Amber Crabtree, Alexandria C. Murphy, Brenita C. Jenkins, Praveena Prasad, Chantell Evans, Brittany Taylor, Margaret Mungai, Mason Killion, Dominique Stephens, Trace A. Christensen, Jacob Lam, Benjamin Rodriguez, Mark A. Phillips, Nastaran Daneshgar, Ho‐Jin Koh, Alice Koh, Jamaine Davis, Nina Devine, Mohammad Saleem, Estevão Scudese, Kenneth Ryan Arnold, Valeria Vanessa Chavarin, Ryan Daniel Robinson, Moumita Chakraborty, Jennifer A. Gaddy, Mariya T. Sweetwyne, Genesis Wilson, Elma Zaganjor, James Kezos, Cristiana Dondi, Anilkumar K. Reddy, Brian Glancy, Annet Kirabo, Anita M. Quintana, Dao‐Fu Dai, Karen Ocorr, Sandra A. Murray, Steven M. Damo, Vernat Exil, Blake Riggs, Bret C. Mobley, Jose A. Gomez, Melanie R. McReynolds, Antentor Hinton

**Affiliations:** ^1^ Department of Molecular Physiology and Biophysics Vanderbilt University Tennessee Nashville USA; ^2^ Department of Internal Medicine University of Iowa Iowa Iowa City USA; ^3^ National Heart, Lung and Blood Institute, National Institutes of Health Maryland Bethesda USA; ^4^ Central Microscopy Research Facility University of Iowa Iowa Iowa City USA; ^5^ Department of Biochemistry and Molecular Biology, The Huck Institute of the Life Sciences Pennsylvania State University Pennsylvania State College USA; ^6^ Department of Cell Biology Duke University School of Medicine North Carolina Durham USA; ^7^ J. Crayton Pruitt Family Department of Biomedical Engineering University of Florida Florida Gainesville USA; ^8^ Microscopy and Cell Analysis Core Facility Mayo Clinic Minnesota Rochester USA; ^9^ Department of Integrative Biology Oregon State University Oregon Corvallis USA; ^10^ Department of Biological Sciences Tennessee State University Tennessee Nashville USA; ^11^ Department of Medicine Vanderbilt University Medical Center Tennessee Nashville USA; ^12^ Department of Biochemistry, Cancer Biology, Neuroscience, and Pharmacology Meharry Medical College Tennessee Nashville USA; ^13^ Laboratory of Biosciences of Human Motricity (LABIMH) of the Federal University of State of Rio de Janeiro (UNIRIO) Rio de Janeiro Brazil; ^14^ Sport Sciences and Exercise Laboratory (LaCEE) Catholic University of Petrópolis (UCP) Petrópolis State of Rio de Janeiro Brazil; ^15^ Department of Ecology and Evolutionary Biology University of California at Irvine California Irvine USA; ^16^ Department of Medicine Health and Society Vanderbilt University Tennessee Nashville USA; ^17^ Department of Pathology, Microbiology and Immunology Vanderbilt University Medical Center Tennessee Nashville USA; ^18^ Department of Veterans Affairs Tennessee Valley Healthcare Systems Tennessee Nashville USA; ^19^ Department of Laboratory Medicine and Pathology University of Washington Washington Seattle USA; ^20^ Sanford Burnham Prebys Medical Discovery Institute California La Jolla USA; ^21^ Department of Medicine Baylor College of Medicine Texas Houston USA; ^22^ National Institute of Arthritis and Musculoskeletal and Skin Diseases, National Institutes of Health Maryland Bethesda USA; ^23^ Department of Biological Sciences, Border Biomedical Research Center University of Texas at El Paso Texas El Paso USA; ^24^ Department of Pathology University of Johns Hopkins School of Medicine Maryland Baltimore USA; ^25^ Department of Cell Biology, School of Medicine University of Pittsburgh Pennsylvania Pittsburgh USA; ^26^ Department of Life and Physical Sciences Fisk University Tennessee Nashville USA; ^27^ Center for Structural Biology Vanderbilt University Tennessee Nashville USA; ^28^ Department of Pediatrics, Carver College of Medicine University of Iowa Iowa Iowa City USA; ^29^ Department of Pediatrics, Division of Cardiology St. Louis University School of Medicine Missouri St. Louis USA; ^30^ Department of Biology San Francisco State University California San Francisco USA; ^31^ Department of Pathology Vanderbilt University Medical Center Tennessee Nashville USA

**Keywords:** 3D morphometry, aging, *Drosophila*, MICOS, mitochondria, mitochondrial disease, mitochondrion, reconstruction, reticulum, serial block‐face SEM, skeletal muscle

## Abstract

During aging, muscle gradually undergoes sarcopenia, the loss of function associated with loss of mass, strength, endurance, and oxidative capacity. However, the 3D structural alterations of mitochondria associated with aging in skeletal muscle and cardiac tissues are not well described. Although mitochondrial aging is associated with decreased mitochondrial capacity, the genes responsible for the morphological changes in mitochondria during aging are poorly characterized. We measured changes in mitochondrial morphology in aged murine gastrocnemius, soleus, and cardiac tissues using serial block‐face scanning electron microscopy and 3D reconstructions. We also used reverse transcriptase‐quantitative PCR, transmission electron microscopy quantification, Seahorse analysis, and metabolomics and lipidomics to measure changes in mitochondrial morphology and function after loss of mitochondria contact site and cristae organizing system (MICOS) complex genes, *Chchd3*, *Chchd6*, and *Mitofilin*. We identified significant changes in mitochondrial size in aged murine gastrocnemius, soleus, and cardiac tissues. We found that both age‐related loss of the MICOS complex and knockouts of MICOS genes in mice altered mitochondrial morphology. Given the critical role of mitochondria in maintaining cellular metabolism, we characterized the metabolomes and lipidomes of young and aged mouse tissues, which showed profound alterations consistent with changes in membrane integrity, supporting our observations of age‐related changes in muscle tissues. We found a relationship between changes in the MICOS complex and aging. Thus, it is important to understand the mechanisms that underlie the tissue‐dependent 3D mitochondrial phenotypic changes that occur in aging and the evolutionary conservation of these mechanisms between *Drosophila* and mammals.

## Graphical abstract

These studies have provided a more comprehensive understanding of the structural and functional changes associated with aging and loss of the MICOS complex. Together, this verifies there is an age‐related loss of both mitochondria structure and the MICOS complex, and shows that the MICOS complex affects the mitochondrial structure, thus suggesting that the MICOS complex plays an evolutionarily conserved role in age‐dependent mitochondrial structural and functional decline.

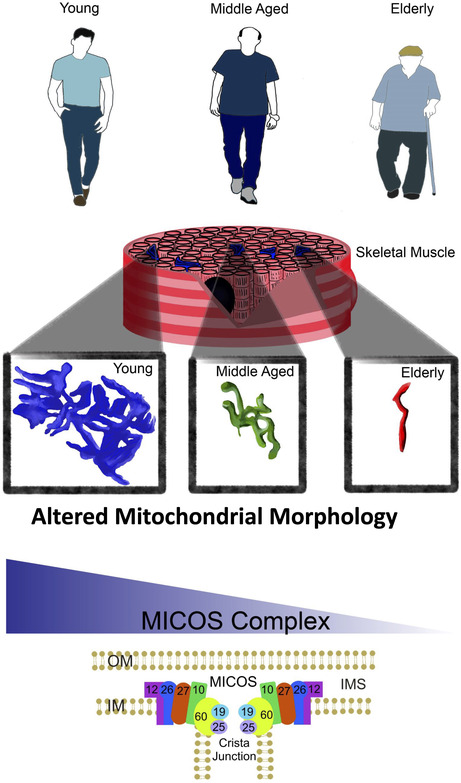

## 1 INTRODUCTION

Sarcopenia, the loss of muscle mass associated with aging and decreased quality of life, affects primarily type II muscle fibers but also type I fibers. With age, sarcopenia in skeletal muscle leads to a body mass‐independent loss of skeletal function (Miller et al., 2019). Mitochondrial dysfunction and alterations in mitochondrial structure are also hallmarks of aging (Haas, 2019). The decreased expression of genes associated with mitochondrial dynamics and the loss of function contribute to sarcopenia and other age‐related diseases (Coen et al., 2019). Thus, mitochondria are a key target for the development of therapeutics for age‐related pathologies (Coen et al., 2019; Haas, 2019). Mitochondria change dynamically, using fission and fusion to tightly regulate structures that are critical to their function (Anand et al., 2014; Cogliati et al., 2016; Kühlbrandt, 2015); therefore, it is important to understand changes in mitochondrial structure over time. The cristae, the inner folds of the mitochondrial membrane, carry out oxidative phosphorylation and contain various transporters (Cogliati et al., 2016). To test our hypothesis that age‐related alterations in metabolism and lipids increase mitochondrial fragmentation and loss of cristae integrity, we determined how mitochondrial structure changes during aging.

Disruption of optic atrophy 1 (OPA‐1), an inner membrane protein that regulates mitochondrial fusion, causes mitochondrial fragmentation and affects the dimensions, shapes, and sizes of the cristae (Cogliati et al., 2016), and disruption of dynamin‐related protein‐1 (DRP1), which is associated with mitochondrial fission, causes elongated mitochondria and resistance to cristae remodeling (Favaro et al., 2019; Otera et al., 2013). Nanotunnels, or “mitochondria‐on‐a‐string,” are thin, double‐membrane protrusions that allow mitochondria to communicate across distances. Nanotunnels, which may increase in mitochondrial disease (Vincent et al., 2017; Zhang et al., 2016), may also be associated with mitochondrial dysfunction during aging. Thus, the concomitant changes in mitochondrial structure and bioenergetics may drive pathologies.

Mutations in genes that regulate the morphology of cristae are associated with aging cardiomyocytes (Zhang, He, et al., 2021). These proteins, located at the crista junctions in the inner membrane, are part of the mitochondrial contact site and cristae organizing system (MICOS) complex, which is important for maintaining mitochondrial shape and size (Kozjak‐Pavlovic, 2017). Loss of DRP1 or OPA‐1 affects mitochondrial morphology similarly (Garza‐Lopez et al., 2022; Lam et al., 2021). Cristae membranes contain the electron transport chain complexes and ATP synthases for oxidative phosphorylation (Friedman et al., 2015; Hu et al., 2020; Rampelt et al., 2017). Because mitochondrial morphology affects function, altering the structure by knocking out MICOS‐associated genes or OPA‐1, a GTPase, may affect mitochondrial function during aging (Friedman et al., 2015; Hu et al., 2020; Rampelt et al., 2017). We hypothesize that MICOS‐associated proteins are lost during aging and that loss of MICOS‐associated genes can mimic the age‐associated changes in mitochondrial morphology. Therefore, we determined how the MICOS complex affects gross mitochondrial structure, beyond the cristae, as well as how mitochondrial structure changes in aging.

Mitochondria have a tissue‐dependent response to the environment (Holmström et al., 2012), which may be due to heterogeneity in mitochondrial DNA (mtDNA) quality check mechanisms across different tissues (Herbers et al., 2019). The 3D reconstruction of tissues using manual contour tracings provides information on mitochondrial phenotypes and how they differ across tissue types. To better understand age‐related changes in mitochondrial structure, we used 3D reconstructions of aged gastrocnemius, soleus, and cardiac tissue in 3‐month‐old and 2‐year‐old mice to compare the size, shape, quantity, complexity, and branching of mitochondria. We observed an age‐related loss of transcripts of the MICOS complex. We also used CRISPR/Cas9 on myotubes to knockout three genes of the MICOS complex, *Chchd3* (Mic19), *Chchd6* (Mic25), and *Mitofilin* (Mic60), to determine whether loss of the MICOS complex may be phenotypically similar to aging through modulation of mitochondrial size, morphology, and oxygen consumption rate. To further characterize factors affecting mitochondrial structure in aging, we used multivariate analysis to identify changes in metabolites. We also characterize the lipidome during aging to identify possible commonalities in metabolic changes that occurred with the loss of the MICOS complex. Finally, we also used a *Drosophila* model to better understand the evolutionarily conserved role of the MICOS complex in aging.

## 2 RESULTS

### 2.1 Aging reduces mitochondrial size in murine gastrocnemius, soleus, and cardiac muscles

The gastrocnemius, a mixed muscle with both mitochondria‐rich oxidative fibers and mitochondria‐poor glycolytic fibers (Mukund & Subramaniam, 2020), is ideal for studying changes in mitochondrial dynamics. In contrast, soleus tissue, with predominantly slow‐twitch muscle fibers, relies on mitochondrial oxidative metabolism (Crupi et al., 2018). We characterized mitochondria in cardiac tissue (Vue et al., 2022), which relies on efficient energy transfer to myofibrils and constant ATP production for contractile function (Chaudhary et al., 2011). Because mitochondrial function depends on structure (Cogliati et al., 2016; Kühlbrandt, 2015), it is important to determine how that structure changes over time. We hypothesized that, over time, mitochondrial fragmentation correlates with loss of the integrity of the cristae.

**FIGURE 1:**
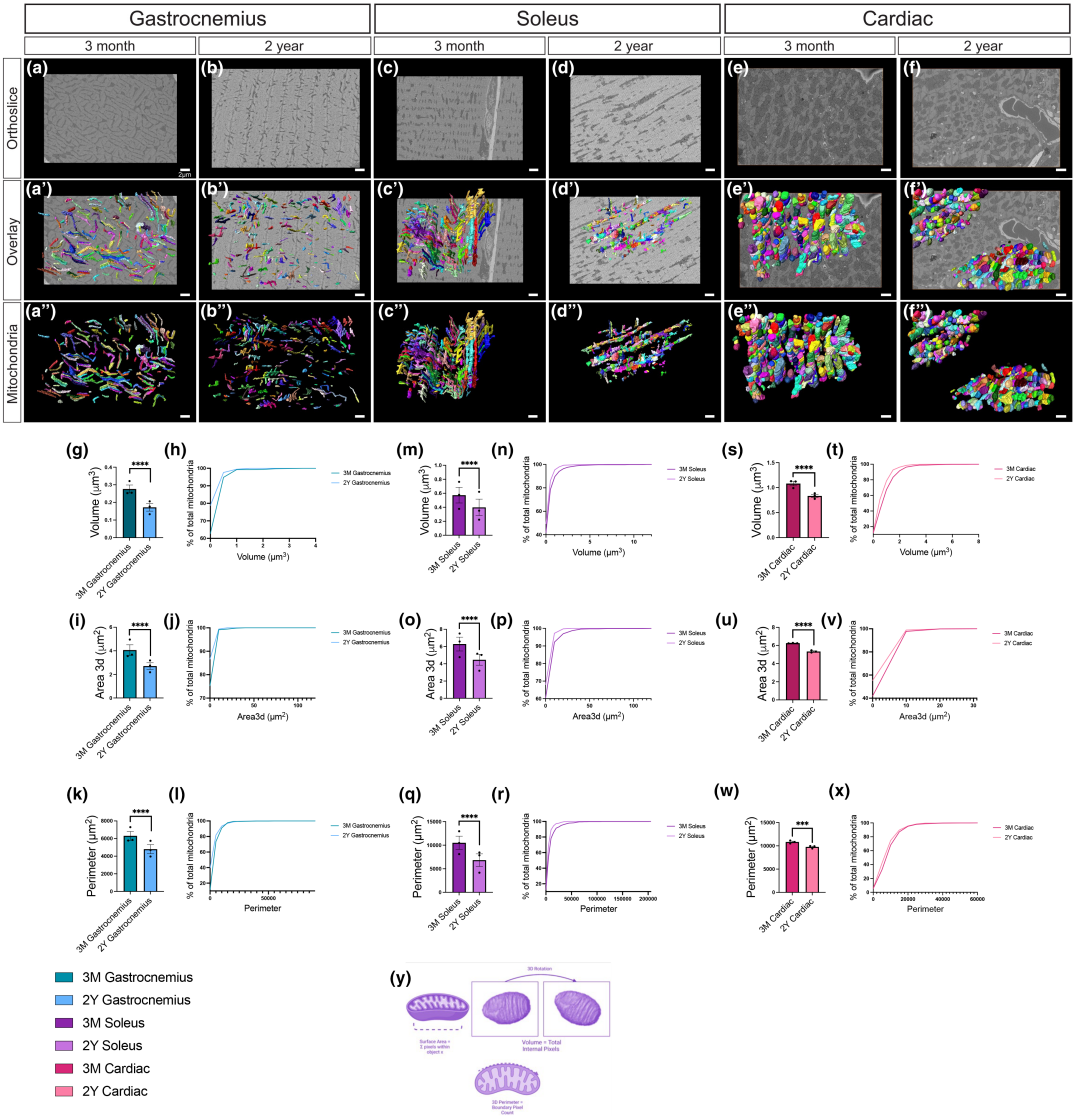
Decreased mitochondrial size and volume in the gastrocnemius, soleus, and cardiac muscle of aged mice in SBF‐SEM 3D reconstructions. (a, b) Representative SBF‐SEM orthoslices for male murine gastrocnemius, (c, d) soleus, and (e, f) cardiac tissues. (a′, b′) 3D reconstructions of mitochondria (various colors) in gastrocnemius, (c′, d′) soleus, and (e′, f′) cardiac tissues from 3‐month‐old and 2‐year‐old mice overlaid on ortho slices. (a″, b″) Pseudo‐colored individual mitochondria in gastrocnemius, (c″, d″) soleus, and (e″, f″) cardiac tissues identify micro‐level changes. (g–x) Quantification of 3D reconstructions, with each dot representing the average for all mitochondria quantified for one mouse. (g) Mitochondrial volume in the gastrocnemius muscle from 3‐month‐old and 2‐year‐old mice and (h) mitochondrial volume distributed as the percent of total mitochondria to visualize relative heterogeneity. (i) Mitochondrial 3D area in gastrocnemius muscle from 3‐month‐old and 2‐year‐old mice and (j) mitochondrial area distributed as the percent of total mitochondria to visualize relative heterogeneity. (k) Mitochondrial perimeter in gastrocnemius muscle from 3‐month‐old and 2‐year‐old mice and (l) mitochondrial perimeter distributed as the percent of total mitochondria to visualize relative heterogeneity. These quantifications are also displayed in (m–r) soleus and (s–x) cardiac tissues. Cartoon representations of metrics to calculate (y) mitochondrial volume, perimeter, and perimeter. Approximately 550 mitochondria were analyzed for each tissue type and age cohort (*n* = 3 mice per age cohort). Significance values: **** represents *p* ≤ 0.0001.

To determine how aging alters mitochondrial networks and individual mitochondrial structures, we imaged gastrocnemius, soleus, and cardiac biopsies from adolescent (3‐month‐old) and aged (2‐year‐old) mice by serial block‐face scanning electron microscopy (SBF‐SEM) with a resolution of 10 nm for the x‐ and y‐planes and 50 nm for the z‐plane, to visualize the electron connectome (Vue Zer et al., 2023). Approximately 50 intermyofibrillar (IMF) mitochondria were segmented from each image stack (Figure 1a–f) to generate a 3D surface view (Figure 1a′–f′). We analyzed IMF mitochondria instead of other mitochondrial subpopulations, such as subsarcolemmal, as IMF mitochondria are larger and display more significant age‐related changes. We analyzed mitochondrial sub‐network volumes from four regions of interest (ROIs) with an average of 175 mitochondria for each mouse (*n* = 3), for a total of over 500 mitochondria. Mitochondrial networks in skeletal muscle tissue in aged mice showed largely interconnected mitochondria (Figure 1a″–d″). As in our previous study (Vue et al., 2022), we found cardiac tissue mitochondria remained relatively clumped with no apparent changes in their distribution (Figure 1e″–f″). We found that across all tissue types, the volume, area, and perimeter of mitochondria were significantly lower for 2‐year‐old versus 3‐month‐old mouse samples (Figure 1g–x). The mitochondrial volume is a measure of total capacity the area is analogous to surface area and the perimeter represents the boundary pixel count (Figure 1aa).

Although there was some variability among the three mice for each age cohort (Figure S1), this heterogeneity was more pronounced in the gastrocnemius (Figure 1g,h). The gastrocnemius also showed a greater reduction in mitochondrial size and surface area, with much smaller mitochondria that lacked hyperbranching. We found similar heterogeneity between the samples of soleus tissue and reductions in mitochondrial volume with aging (Figure 1m–r). In contrast to skeletal muscle, the mitochondria were more homogenous in cardiac tissue (Figure 1s–x). Overall, in older mice, we saw a decrease in mitochondrial volume, area, and perimeter that was associated with increased fragmentation and smaller mitochondria. Because the size and length of mitochondria decreased with age, we further characterized the complexity of the mitochondria, which is implicated in mitochondrial communication.

### 2.2 Aging results in poorly connected mitochondria with decreased branching in murine gastrocnemius, soleus, and cardiac muscles

**FIGURE 2:**
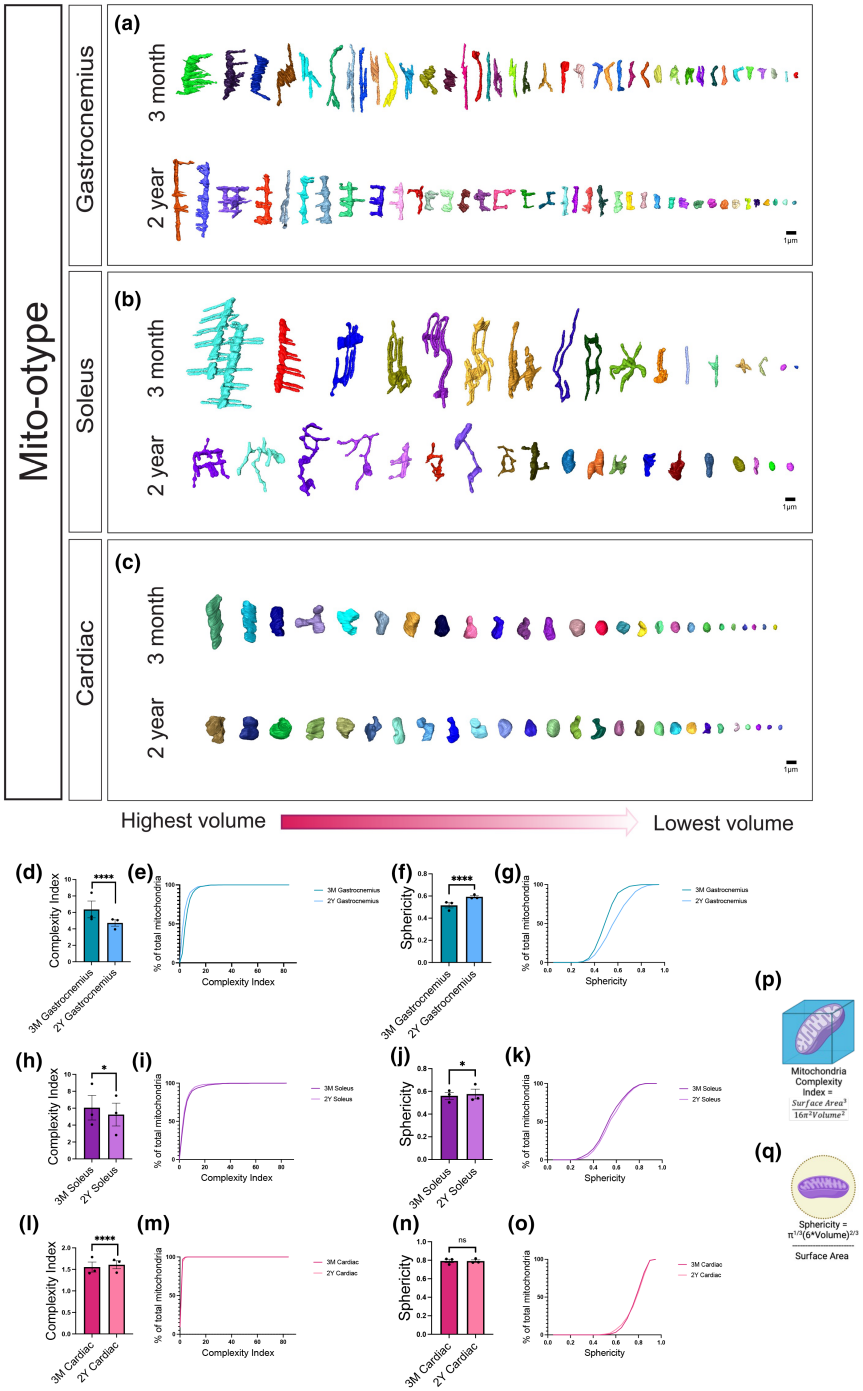
SBF‐SEM 3D reconstruction in gastrocnemius, soleus, and cardiac muscle of aged mice showed altered mitochondrial networks. Representative examples of 3D reconstruction of mitochondria in (a) gastrocnemius, (b) soleus, and (c) cardiac tissue of 3‐month‐old and 2‐year‐old mice organized by volume to show the mitochondrial phenotypes. (d) Mitochondrial complexity index (MCI), analogous to sphericity, in the gastrocnemius muscle from 3‐month‐old and 2‐year‐old mice, and (e) MCI distributed as the percent of total mitochondria to visualize relative heterogeneity. (f) Sphericity in the gastrocnemius muscle from 3‐month‐old and 2‐year‐old mice and (g) mitochondrial sphericity distributed as the percent of total mitochondria to visualize relative heterogeneity. These quantifications are also displayed in (h–k) soleus and (l–o) cardiac tissues. Cartoon representations of metrics to calculate (p) MCI and (q) sphericity. Approximately 550 mitochondria were analyzed for each tissue type and age cohort (*n* = 3 mice per age cohort). Significance values: *p ≤ 0.05; ****p ≤ 0.0001.

We hypothesized that fewer networks and simpler shapes would occur with aging and dysfunction; therefore, we measured mitochondrial complexity to identify changes in mitochondrial shape during aging. Because we observed in 3D reconstructions that mitochondrial populations are heterogeneous and diverse, we used mito‐otyping, a karyotyping‐like method for arranging mitochondria (Vincent et al., 2019), to visualize the diversity of IMF mitochondria (Figure 2a–c). We found, for every volumetric measurement, smaller and less complex mitochondria with age. Soleus and gastrocnemius tissues showed more branched mitochondria in the adolescent mice versus the aged mice, and the latter had smaller volumes. In contrast to cardiac tissue, skeletal muscle showed large phenotypic changes with aging. To validate these changes, we analyzed 3D mitochondrial complexity using 3D form‐factor measurements (Koopman et al., 2005; Vincent et al., 2019). We measured the mitochondrial complexity index (MCI) and sphericity to further characterize changes in complexity (Figure 2d–o). MCI and sphericity measure the roundness of mitochondria (Figure 2p,q). In gastrocnemius tissue, MCI increased concomitantly with sphericity in aged mice (Figure 2d–g). Thus, in contrast to their appearance in mito‐otyping, in aged mice, mitochondria in the gastrocnemius showed increased sphericity. Among the young and the old mice, there were variations for both metrics. Soleus tissue, which showed a similar heterogeneity, was less complex and more spherical in aged mice compared to gastrocnemius tissue (Figure 2h–k), although this was less significant than in the gastrocnemius. Finally, cardiac tissue showed a significant increase only in MCI (Figure 2l–o). Together, these data suggest that complexity changes with age but is tissue‐type dependent. Therefore, we determined the role of the MICOS complex in age‐related changes in mitochondrial structure and function.

### 2.3 Age‐related changes in MICOS complex mRNA transcripts

**FIGURE 3:**
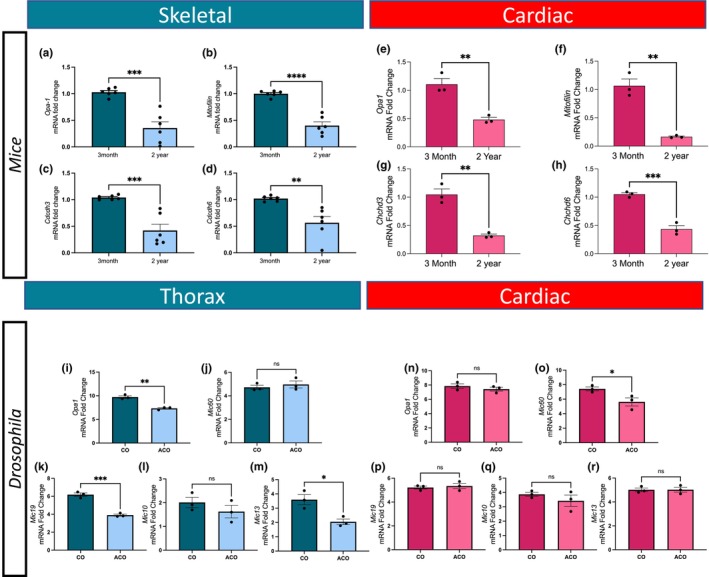
Changes in mRNA transcripts of *Opa1* and MICOS genes in aged mouse tissue and *Drosophila*. (a–d) Fold changes in the amount of *Opa1* and MICOS gene transcripts in mitochondria of skeletal muscle from 3‐month‐old and 2‐year‐old mice as measured by RT‐qPCR. (a) *Opa1*, (b) *Mitofilin*, (c) *Chchd3*, and (d) *Chchd6* transcripts in skeletal muscle. (e–h) Fold changes in transcripts of *Opa1* and MICOS genes in 3‐month‐old and 2‐year‐old murine cardiac tissue. (e) *Opa1*, (f) *Mitofilin*, (g) *Chchd3*, (h) and *Chchd6* transcripts. (i–r) Altered *Drosophila* mitochondrial genes with age. Fold changes in transcripts of *Opa1* and MICOS genes in aging *Drosophila* in (i) *Opa1*, (j) *Mic60* (*Mitofilin*), (k) *Mic19* (*Chchd3*), (l) *Mic10* (MICOS10), and (m) *Mic13* (QIL1) transcripts in thorax. Fold changes in transcripts of *Opa1* and MICOS genes in aging *Drosophila* in (n) *Opa1*, (o) *Mic60* (*Mitofilin*), (p) *Mic19* (*Chchd3*), (q) *Mic10* (MICOS10), and (r) *Mic13* (QIL1) transcripts in cardiac tissue. Significance values: *p ≤ 0.05; **p ≤ 0.01; ***p ≤ 0.001; ****p ≤ 0.0001. For all RT‐qPCR experiments, *n* = 6.

Although the MICOS complex and OPA‐1 are key players in mitochondrial biogenesis (Hu et al., 2020; Kozjak‐Pavlovic, 2017; Li et al., 2016), how their interactions regulate aging and mitochondrial structures is poorly understood. Further, aging causes mitochondrial fragmentation (Haas, 2019), which is associated with the loss of *Opa1* and MICOS complex proteins (Genin et al., 2016, 2018; Hu et al., 2020). Thus, we hypothesized that aging decreases the MICOS complex proteins. Therefore, we measured transcripts for four principal MICOS complex subunit genes, *Opa1*, *Chchd3* (*Mic19*), *Chchd6* (*Mic25*), and *Mitofilin* (*Mic60*) using reverse transcriptase qPCR (RT‐qPCR). Based on prior studies that found loss of *Opa1* during aging, we used *Opa1* as a positive control (Hu et al., 2020; Khin et al., 2021; Varanita et al., 2015; Zheng et al., 2019). We found a significant loss of transcripts for *Opa1* and MICOS complex subunit genes in aged versus young skeletal (Figure 3a–d) and cardiac (Figure 3e–h) tissue.

We further extended our aging studies by comparing transcript levels for MICOS complex genes and mitochondrial endoplasmic reticulum contact (MERC) genes in experimentally evolved *Drosophila* populations subjected to hundreds of generations of accelerated aging versus control flies (Phillips et al., 2022). Although the relationship between the MICOS complex and MERCs is unclear, we thought there could be a concomitant decrease in MERC and MICOS complex gene expression. We compared expression patterns between control (CO) flies and accelerated aging flies (ACO), which show large differences in age‐specific mortality, gene expression profiles, and metabolomic profiles (Barter et al., 2019; Burke et al., 2016; Phillips et al., 2022). In flight muscle tissues, ATF‐4, Drp, Marf, Opa1, MINOS1 (*Mic10*), CHCHD3/6 (*Mic19*), QIL1 (*Mic13*), APOO (*Mic16/27*), IP3R/Itpr, VDAC/Porin, Bip (*Grp78*), GRP‐75/Hsc70‐5, and Ire1 were all downregulated in the indirect flight muscle of aged flies compared with controls (Table 1; Figure 3i–r). However, only the Drp, Marf, Opa1, CHCHD3/6, QIL1, APOO, IP3R, VDAC, and Bip changes were significant (*p*‐value < 0.05; unpaired *t*‐test) (Table 1; Figure 3i–m). The small increases in ATF‐6 and Mitofilin expression in the aged flies were not significant. In the cardiac tissues of aged flies, ATF‐4, Opa1, MINOS1, and Mitofilin were downregulated compared with controls; however, only the changes in ATF‐4 and Mitofilin expression were significant (Table 2; Figure 3n–r). In contrast, FGF‐21 expression increased significantly in the aged flies (Table 2). These support the results in mice showing that mitochondrial, MICOS, and MERC mRNA transcripts respond differently in cardiac and skeletal muscle tissue during aging, but in some organisms, the MICOS complex shows greater changes during aging.

**TABLE 1:** *Drosophila* RT‐qPCR flight tissue data for knockdowns of the indicated genes for MICOS, mitochondrial, and MERCs proteins during aging.

Gene	∆∆*C*_t_	Fold change
*ATF‐4*	−0.781	1.718
*Drp*	−1.575	2.98*
*Marf*	−1.764	3.397*
*Opa1*	−2.388	5.235*
*Dmic60 (mitofilin)*	0.244	0.844
*CHCHD3/6 (mic19)*	−2.279	4.853*
*MINOS1 (Mic10)*	−0.385	1.306
*QIL1 (Mic13)*	−1.549	2.926*
*APOO (mic26/27)*	−1.053	2.075*
*IP3R/Itpr*	−0.997	1.996*
*VDAC/porin*	−3.265	9.612*
*FGF21/bln*	−0.14	1.102
*ATF‐6*	0.072	0.952
*Bip(grp78)/Hsc 70‐3*	−2.259	4.786*
*GRP75/Hsc70‐5*	−1.203	2.301
*Ire1*	−1.219	2.328

*Note*: The fold change compares expression in the ACO population versus the CO population. Fold‐change values of >1.0 indicate that the gene is expressed more in the CO populations (28‐day cycle) than in the ACO populations (9‐day cycle) at Day 21. Fold‐change values of <1.0 indicate that the gene is expressed more in the ACO populations than in the CO populations at Day 21. Values of ~1.0 indicate no difference between the populations. Statistically significant fold‐change values are denoted by an asterisk (*).

**TABLE 2:** RT‐qPCR results from *Drosophila* cardiac tissue.

Gene	∆∆*C*_t_	Fold change
*ATF‐4*	−0.595	1.51*
*Drp*	0.01	0.993
*Marf*	0.032	0.978
*Opa1*	−0.428	1.346
*MINOS1 (Mic10)*	−0.452	1.368
*QIL1 (Mic13)*	0.012	0.992
*CHCHD3/6 (mic19)*	0.123	0.918
*Dmic60 (mitofilin)*	−1.791	3.46*
*APOO (mic26/27)*	−0.012	1.008
*VDAC/Porin*	0.29	0.818
*IP3R/Itpr*	0.243	0.845
*FGF21/bln*	0.927	0.526*
*Ire1*	0.221	0.858
*Bip(grp78)/Hsc 70‐3*	−0.223	1.167
*ATF‐6*	0.141	0.907
*GRP75/Hsc70‐5*	−0.224	1.168

*Note*: *Drosophila* RT‐qPCR cardiac tissue data for knockdowns of the indicated genes for MICOS, mitochondrial, and MERCs proteins during aging. The fold change compares expression in the ACO population versus the CO population. Fold‐change values of >1.0 indicate that the gene is expressed more in the CO populations (28‐day cycle) than in the ACO populations (9‐day cycle) at Day 21. Fold‐change values of <1.0 indicate that the gene is expressed more in the ACO populations than in the CO populations at Day 21. Values of ~1.0 indicate no difference between the populations. Statistically significant fold‐change values are denoted by an asterisk (*).

### 2.4 2D and 3D structural changes in cristae and mitochondria after loss of the MICOS complex and *Opa1*

To determine the role of OPA‐1 and the MICOS complex in mitochondrial structure and networking, we ablated the genes for *Opa1* and the MICOS complex proteins in isolated primary skeletal muscle cells from 3‐month‐old mice. We isolated primary satellite cells and then differentiated myoblasts into myotubes. Using a CRISPR/Cas9 method and a control plasmid, we knocked out the genes for MICOS complex components and *Opa1* from skeletal muscle cells.

**FIGURE 4:**
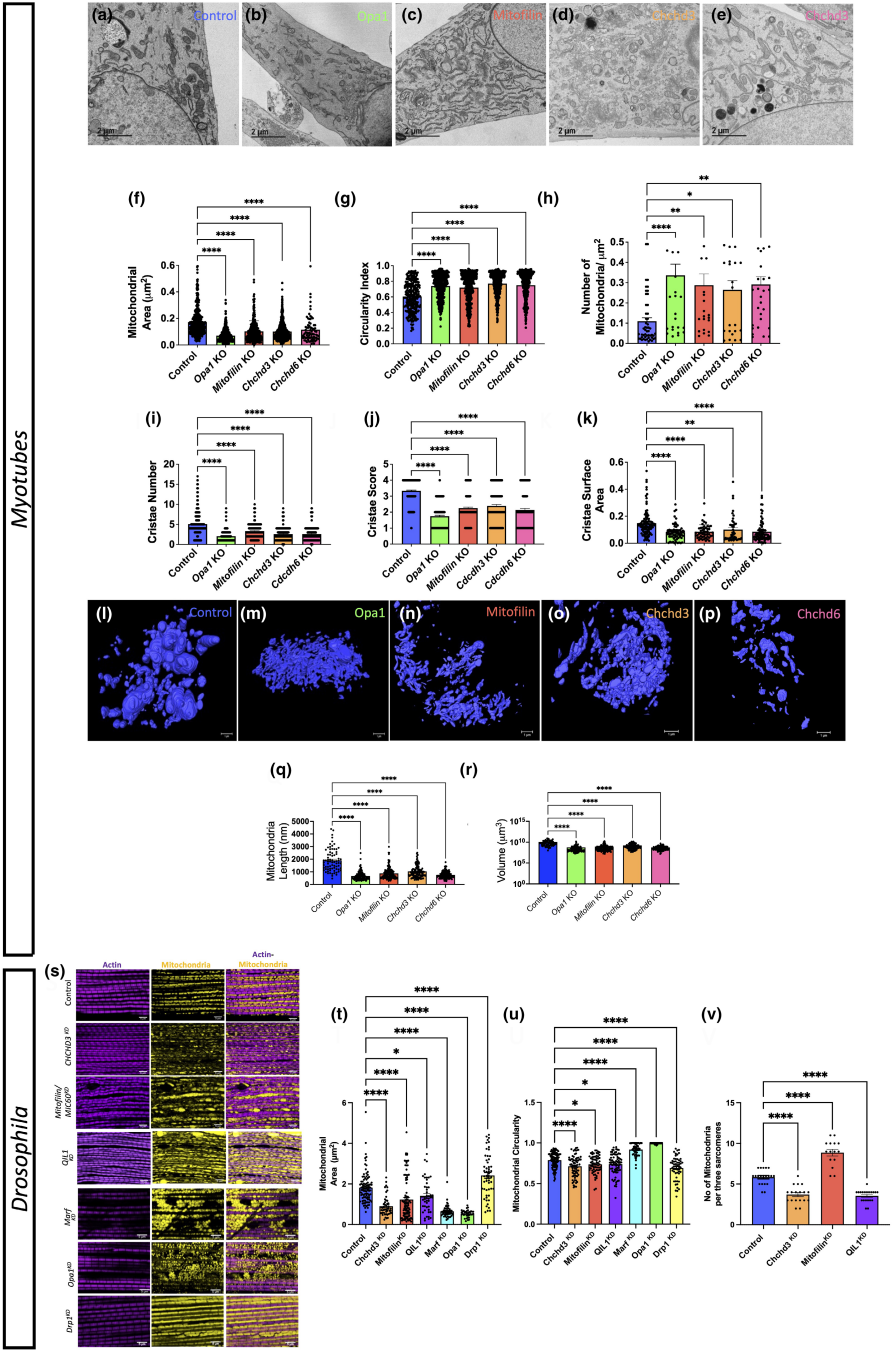
Knockout of *Opa1*, *Mitofilin*, *Chchd3*, or *Chchd6* in myotubes resulted in structural changes of mitochondria and cristae in TEM and 3D reconstruction. (a–e) Representative images of mitochondria and cristae from myotubes of *Opa1*, *Mitofilin*, *Chchd3*, and *Chchd6* knockout mice compared to WT. (f) Mitochondrial area in myotubes of *Opa1*, *Mitofilin*, *Chchd3*, and *Chchd6* knockout mice compared to WT. (g) Circularity index, measuring the roundness and symmetry of mitochondria, in myotubes of *Opa1*, *Mitofilin*, *Chchd3*, and *Chchd6* knockout mice compared to WT. (h) The number of mitochondria in myotubes of *Opa1*, *Mitofilin*, *Chchd3*, and *Chchd6* knockout mice compared to WT. (i) The number of individual cristae in myotubes of *Opa1*, *Mitofilin*, *Chchd3*, and *Chchd6* knockout mice compared to WT. (j) Cristae scores measuring the uniformity and idealness of cristae in myotubes of *Opa1*, *Mitofilin*, *Chchd3*, and *Chchd6* knockout mice compared to WT. (k) The surface area of the average cristae in myotubes of *Opa1*, *Mitofilin*, *Chchd3*, and *Chchd6* knockout mice compared to WT. (l–p) Representative images showing 3D reconstructions of mitochondria in myotubes of *Opa1*, *Mitofilin*, *Chchd3*, and *Chchd6* knockout mice compared to WT. (q) Mitochondrial 3D length in myotubes of *Opa1*, *Mitofilin*, *Chchd3*, and *Chchd6* knockout mice compared to WT. (r) Mitochondrial volume on a log scale in myotubes of *Opa1*, *Mitofilin*, *Chchd3*, and *Chchd6* knockout mice compared to WT. (s–v) Altered *Drosophila* mitochondrial structure resulting from loss of the MICOS complex and mitochondrial proteins. (s) Actin‐mitochondria staining for *Drosophila* flight tissue in knockouts of MICOS complex and mitochondrial proteins. TEM quantification of mitochondrial changes in *Drosophila* flight tissue for (t) mitochondrial area, (u) circularity, and (v) quantity per sarcomere upon knockout of MICOS complex and mitochondrial proteins. Significance values: *p ≤ 0.05; **p ≤ 0.01; ***p ≤ 0.001; ****p ≤ 0.0001. Dots represent the number of mitochondria quantified.

We measured 1250 mitochondria across 10 cells, with loss of *Opa1* as a positive control for mitochondrial morphological changes because in vitro deletion of *Opa1* alters mitochondrial morphology (Hinton et al., 2023; Lam et al., 2021; Pereira et al., 2017). Although *Opa1* expression decreases with age (Tezze et al., 2017), how the loss of the MICOS complex affects mitochondria 3D morphology is poorly understood. However, knockout of the MICOS subunit *Chchd3* results in fragmented mitochondria as the cristae lose their normal structure (Darshi et al., 2011). Similarly, downregulation of *Chchd6*, which is important in maintaining crista structure, results in hollow cristae that lack an electron‐dense matrix, thereby inhibiting ATP production and cell growth (An et al., 2012; Ding et al., 2015; Ott et al., 2015). Using transmission electron microscopy (TEM), we compared mitochondria and cristae in myotubes from wild‐type (WT) and *Opa1*, *Mitofilin*, *Chchd3*, and *Chchd6* knockout myotubes, which are essential for the organization of mitochondrial cristae (Ding et al., 2015; John et al., 2005; Figure 4a–e). Mitochondrial average area decreased for *Opa1*, *Mitofilin*, *Chchd3*, and *Chchd6* knockout myotubes (Figure 4f), whereas the mitochondrial circularity index (the roundness and symmetry of mitochondria) and the number of mitochondria, once normalized, increased (Figure 4g,h). This suggests that mitochondria become smaller, less complex, and more abundant upon loss of the MICOS complex. For *Opa1*, *Chchd3*, *Mitofilin*, and *Chchd6* knockouts, the number of cristae per mitochondrion decreased, as did the cristae score and cristae surface area compared with the WT (Figure 4i–k). Here, the cristae score is defined as follows:

0: No sharply defined cristae are visible.
1: Over 50% of the mitochondrial area lacks cristae.
2: Over 25% of the mitochondrial area lacks cristae.
3: Irregularly shaped cristae are visible, and less than 25% of mitochondria lack cristae over 75% of the mitochondrial area.
4: Many regular‐shaped cristae are visible, and less than 25% of mitochondria lack cristae.

Although the loss of the MICOS complex resulted in similar changes for all of the knockouts, the *Chchd3* knockout showed the least significant changes. Together, these data showed quantitative and structural changes in both mitochondria and cristae upon loss of MICOS proteins.

TEM provides cristae detail but not 3D morphology; therefore, we used SBF‐SEM to analyze the 3D structure of the mitochondria. We measured a total of 200 mitochondria across 10 cells, comparing WT, *Opa1*, *Mitofilin*, *Chchd3*, and *Chchd6* knockout myotubes (Figure 4l–p). We found that unlike the elongated mitochondria in the WT, *Opa1* and MICOS protein knockouts had a much shorter 3D length (Figure 4q). Similarly, the mitochondria of *Chchd3*, *Chchd6*, *Mitofilin*, and *Opa1* knockouts had smaller volumes than the WT (Figure 4r). The 3D reconstruction data, in combination with the prior TEM results, show that mitochondrial dynamics change with the loss of MICOS subunits and mimic the mitochondrial phenotypes observed during aging.

We also determined the effect of the loss of the MICOS complex and other mitochondrial genes on mitochondrial structure in a *Drosophila* model. Because QIL1 (*Mic13*) and CHCHD3/6 (*Mic19*) were downregulated and Mitofilin was slightly upregulated in aging skeletal tissue, we determined how knockdown (KD) of these proteins affected *Drosophila* mitochondrial structure as well as loss of mitochondrial fusion proteins OPA1 and MARF and the fission protein DRP1. Mitochondrial‐actin staining showed differences in mitochondrial organization and relative myofibrillar density among the strains (Figure 4s). Using TEM, we found that in a DRP1 KD, in vivo flight muscle showed increased mitochondrial area, whereas MARF and OPA1 KDs reduced mitochondrial area (Figure 4t). Muscles from strains with a KD of the MICOS complex proteins (*Mic13*, *Mic19*, and *Mic60*) had reduced mitochondrial volume, although this reduction was less severe in *Mic13* (Figure 4t). In considering circularity as a factor in mitochondrial complexity, we found that loss of the MICOS complex, similar to the KD of DRP1, decreased circularity (Figure 4u). In contrast, the loss of mitochondrial fusion proteins increased the circularity of mitochondria. Also, KD of *Mic13* and *Mic19* reduced mitochondria, whereas *Mic60*‐deficiency increased mitochondria (Figure 4v). This suggests that the functions of the MICOS complex and related mitochondrial structure are evolutionarily conserved to some degree, but there are organism‐dependent alterations in associated mitochondrial dynamics and age‐related changes in gene expression.

### 2.5 Changes in oxygen respiration rate and metabolites after loss of the MICOS complex and *Opa1*

**FIGURE 5:**
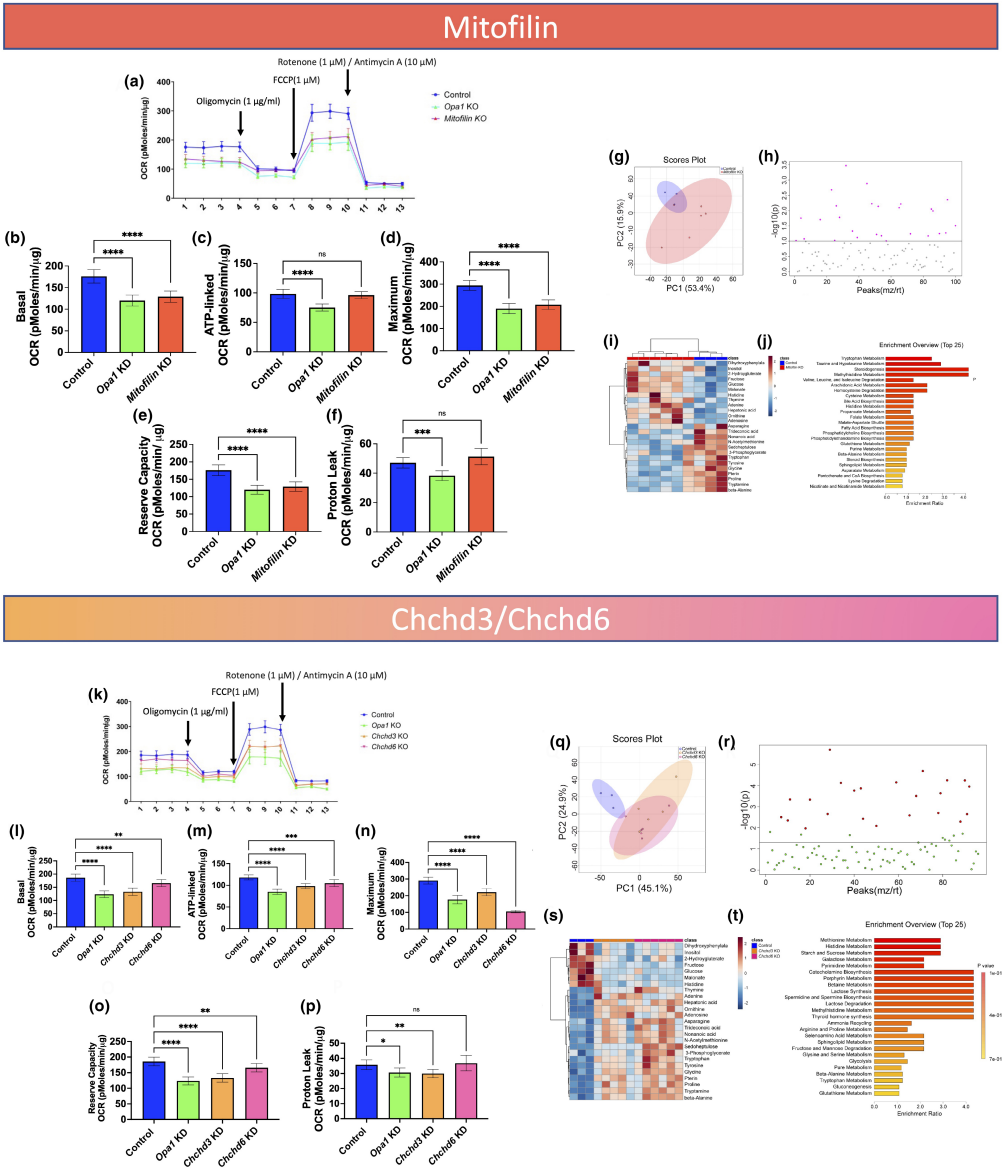
Knockout of the MICOS complex in myotubes resulted in changes in oxygen consumption rates and metabolomics. (a) OCR in myotubes of *Opa1* and *Mitofilin* knockout mice compared to WT. (b) Basal OCR, the net respiration rate once non‐mitochondrial respiration has been removed, in myotubes of *Opa1* and *Mitofilin* knockout mice compared to WT. (c) ATP‐dependent respiration, shown from intervals 4–7 in the OCR graphs, was determined by the addition of oligomycin (an inhibitor of respiration) in myotubes of *Opa1* and *Mitofilin* knockout mice compared to WT. (d) Maximum OCR represented by the peak from intervals 7–11 once non‐mitochondrial respiration was deducted, in myotubes of *Opa1* and *Mitofilin* knockout mice compared to WT. (e) The reserve capacity, the difference between basal OCR and maximum OCR, in myotubes of *Opa1* and *Mitofilin* knockout mice compared to WT. (f) Proton leak, representing non‐phosphorylating electron transfer, in myotubes of *Opa1* and *Mitofilin* knockout mice compared to WT. (g–j) Metabolomic analysis in *Mitofilin* knockout mice. (g) Metabolite *PCA* and (h) *T*‐test comparing myotubes for control versus *Mitofilin* knockout mice. (i) Heatmap showing the relative abundance of ions and (j) enrichment analysis of metabolites, which links similarly functioning metabolites with the relative abundance for the *Mitofilin* knockout. (k) OCR in myotubes of *Chchd3*, *Chchd6*, and *Opa1* knockout mice compared to WT. (l) Basal OCR in myotubes of *Chchd3*, *Chchd6*, and *Opa1* knockout mice compared to WT. (m) ATP‐linked respiration in myotubes of *Chchd3*, *Cchchd6*, and *Opa1* knockout mice compared to WT. (n) Maximum OCR in myotubes of *Chchd3*, *Chchd6*, and *Opa1* knockout mice compared to WT. (o) The reserve capacity in myotubes of *Chchd3*, *Chchd6*, and *Opa1* knockout mice compared to WT. (p) Proton leak in myotubes of *Opa1*, *Chchd3*, and *Chchd6*, knockout mice compared to WT. (q–t) Metabolomic analysis in *Chchd3* or *Chchd6* knockout mice. (q) Metabolite *PCA* and (r) ANOVA test comparing control to myotubes of *Chchd3* and *Chchd6* knockout mice (s) Heatmap showing the relative abundance of ions for control and (t) enrichment analysis metabolites for *Chchd3* and *Chchd6* knockout mice. Significance values: *p ≤ 0.05; **p ≤ 0.01; ***p ≤ 0.001; ****p ≤ 0.0001. For Seahorse analysis, *n* = 6 plates for experimental knockouts and *n* = 16 for controls.

Loss of *Opa1* induces bioenergetic stress and decreases electron transport chain function (Pereira et al., 2017), and ablation of the MICOS complex alters mitochondrial capacity (Kondadi et al., 2019; Stephan et al., 2020). To determine the effect of the loss of the MICOS complex on mitochondrial function, we measured the oxygen consumption rate (OCR) using an XF24 Seahorse analyzer. We found that loss of *Opa1* or *Mitofilin* in myotubes decreased basal OCR (Figure 5a,b) and decreased ATP‐linked, maximum, and reserve capacity OCR (Figure 5c–e). Although *Opa1* knockout myotubes exhibited a decrease in proton leak, which represents protons that go from the mitochondria to the matrix without producing ATP, *Mitofilin* knockouts showed no significant differences compared to the control (Figure 5f). To determine the global effects of loss of *Opa1* or the MICOS complex in skeletal muscle myotubes, we analyzed the metabolome to identify changes in metabolites that occurred with changes in mitochondria and cristae. Principal component analysis (PCA) revealed distinct populations in the control versus the *Mitofilin* knockout strains, which suggested that their genotypes contributed to the clustering (Figure 5g). To identify the metabolites that best discriminated between true versus false positives, we constructed a model using analysis of variance (ANOVA) to determine the key metabolites that were statistically significant (Figure 5h). This unique metabolite signature revealed that Mitofilin plays a critical role in regulating amino acid metabolism and steroidogenesis (Figure 5i,j). Upregulation of steroidogenesis pathways may result from the increased fluidity of membranes caused by Mitofilin (Kitajima & Ono, 2016; Torres et al., 2018).

In *Opa1*, *Chchd3*, and *Chchd6* knockouts, there was a decrease in basal, ATP‐linked, maximum, and reserve capacity OCR compared with the control (Figure 5k–o). Although proton leak OCR decreased in *Opa1* and *Chchd3* knockout myotubes (Figure 5p), there was no significant difference between the control and *Chchd6*. The decrease in OCR may be attributed to smaller, fragmented mitochondria; mitochondrial density decreases as fragmentation targets them for autophagy (Tezze et al., 2017; Wang et al., 2020). Together, these results showed that MICOS and *Opa1* are essential for the normal respiration of muscle tissue. We also measured the effect of knocking out the genes for *Chchd3* and *Chchd6* in skeletal muscle myotubes on bioenergetic metabolism. PCA revealed distinct populations in the control and the *Chchd3* and *Chchd6* knockouts, similar to what we observed in *Mitofilin* (Figure 5q). We constructed a model using ANOVA to determine which metabolite changes in *Chchd3* and *Chchd6* knockouts were statistically significant (Figure 5r). There was a loss of protein synthesis and changes in carbohydrate metabolism (Figure 5s,t). Loss of *Opa1* typically favors fatty acid synthesis, so the results showing increased carbohydrate metabolism differ from previous *Opa1* knockout responses (Chao de la Barca et al., 2020; Sarzi et al., 2016; Wasilewski et al., 2012). This atypical response was evident in the increase in lactose and starch synthesis, but there was poor protein turnover, as seen in methionine metabolism (Figure 5t). Because a loss of MICOS complex proteins caused a change in metabolism, we determined whether this metabolic change parallels age‐related changes in gastrocnemius, soleus, and cardiac metabolism.

### 2.6 Metabolomics/lipidomic profiles in gastrocnemius, soleus, and cardiac tissue exhibit altered metabolism during aging

**FIGURE 6:**
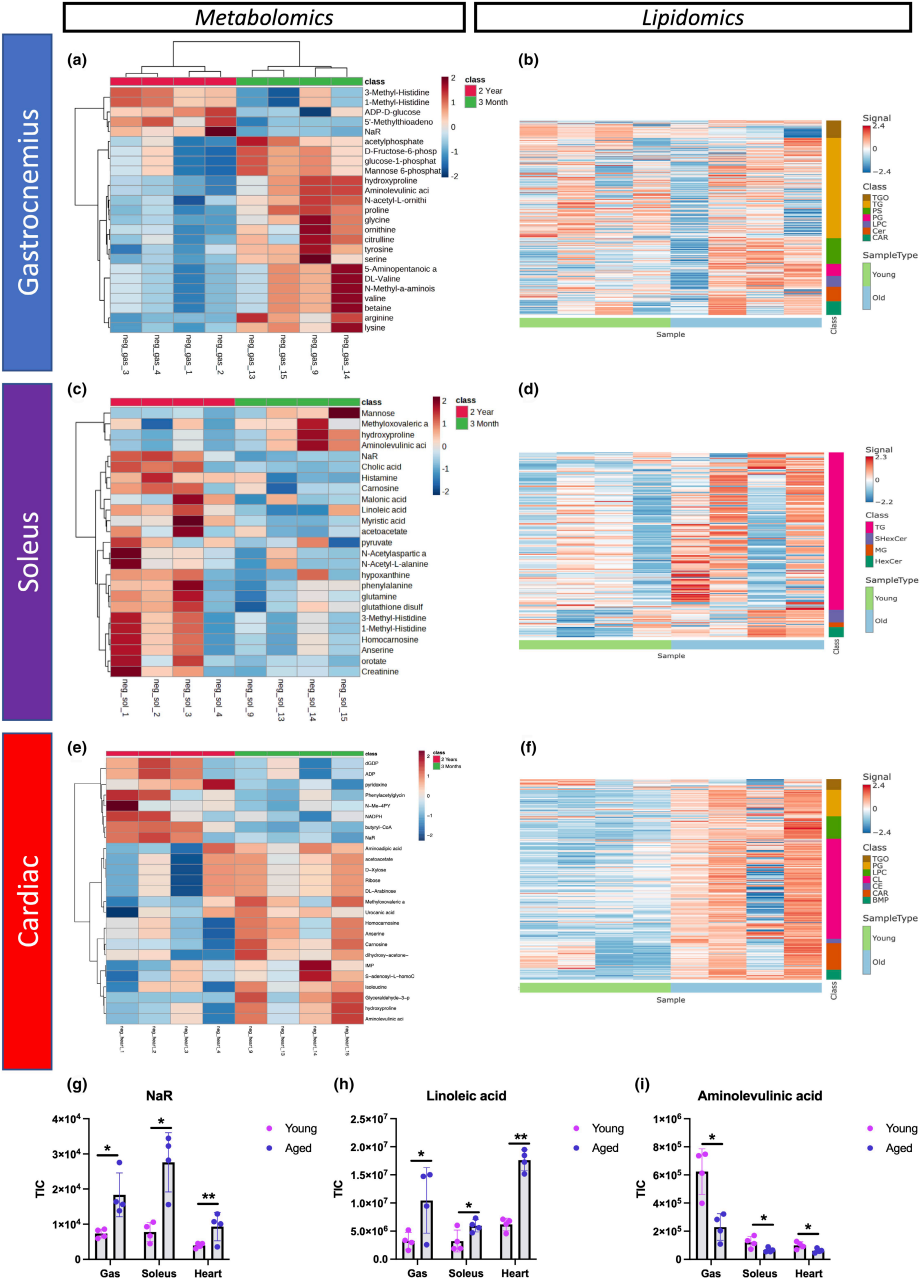
Metabolomics analysis and lipidomic profiling revealed metabolic dysregulation and disruptions in lipid classes with age in gastrocnemius, soleus, and cardiac muscles. (a) Metabolic heatmap showing the relative abundance of metabolites and (b) the lipidome in young and aged gastrocnemius, (c, d) soleus, and (e, f) cardiac samples. For each tissue and metabolite in the heatmaps, the aged samples were normalized to the median of the young samples and then log_2_ transformed. Significantly different lipid classes represented in the figures are those with adjusted *p*‐values < 0.05 (*note*: *p*‐values were adjusted to correct for multiple comparisons using an FDR procedure) and log fold changes greater than 1 or less than −1. Young, *n* = 4; aged, *n* = 4. For all panels, error bars indicate SEM, ** indicates *p* < 0.01; and *p < 0.05, calculated with Student's *t‐*test.

Because loss of the MICOS complex was implicated in altered steroidogenesis and metabolism, we characterized the changes during aging. Using metabolomics and lipidomics analyses on young and aged gastrocnemius, soleus, and cardiac muscle tissues (Figure 6a–f; Figure S2), we found significant metabolic changes in all three tissue types. These changes included various processes, including NAD^+^ metabolism, linolenic acid metabolism, porphyrin synthesis, heme biosynthesis, and glycine and lysine metabolism (Figure S2D–I). Notably, we observed an accumulation of cholic acid in aged soleus and gastrocnemius muscles (Figure S2A,B,D,E), an inducer of muscle atrophy (Abrigo et al., 2021). Amino acid metabolism was dysregulated in aged tissues across all types.

Lipidomic profiling of young versus aged tissues (Figure 6b,d,f; Tables S1–S4) revealed changes in lipid classes (Figure S2J–L) and lipid chain lengths (Figure S2M–O) with age. The greatest changes in lipid classes were in gastrocnemius and cardiac tissues, whereas the soleus showed little dysregulation (Figure S2J–L). Additionally, cardiac muscle showed greater changes in lipid chain lengths than the soleus and gastrocnemius muscles (Figure S2M–O). Notably, altered lipid chain lengths, which affect membrane integrity, fluidity, and functionality, influence a wide range of cellular and physiological processes.

Aged gastrocnemius muscle tissue showed alterations in triglycerides oligomers (TGO), triglycerides (TG), phosphatidylserine (PS), phosphatidylglycerol (PG), ceramide (Cer), and acylcarnitine (CAR) (Figure 6b). In the gastrocnemius, the levels of PS, the most abundant negatively charged phospholipid in eukaryotic membranes and enriched in regions of cell–cell contact (Jeong & Conboy, 2011), decreased with age (Figure S2J). CAR, which transports acyl groups from the cytosol into the mitochondrial matrix for beta‐oxidation (Dambrova et al., 2022), was reduced in aged gastrocnemius tissues (Figure S2J). Conversely, Cer levels increased with age in these tissues (Figure S2J), supporting a previous report that elevated Cer levels in replicative senescent cells contribute to senescence by inducing cell cycle arrest (Stith et al., 2019).

In aged soleus tissues, we observed changes in only TG, sulfatide hexosylceramide (SHexCer), monoacylglycerol (MG), and hexosylceramide (HexCer) (Figure S2K), with decreases in HexCer and SHexCer (Figure S2K). These are sphingolipids, suggesting a novel role for sphingolipids in the aging process of the soleus. MGs, which are linked to a lipotoxicity that triggers immune senescence (Feng et al., 2022), accumulate in the aged soleus, whereas TGs, which are important for storing and transporting fatty acids in cells and in the circulation, decreased in the aged soleus (Figure S2K). These findings align with previous reports of decreased TG levels in plasma and increased levels of fatty acids during aging (Johnson & Stolzing, 2019).

In aged cardiac muscle, we observed changes in TGO, PG, lysophosphatidylcholines (LPC), cardiolipin (CL), cholesteryl esters (CE), CAR, and bis(monoacylglycerol)phosphate (BMP) (Figure S2L). Notably, CL, which plays a critical role in regulating mitochondrial proteins and maintaining mitochondrial structures such as cristae and contact sites (Paradies et al., 2019), was reduced in aged cardiac muscles (Figure S2L). This lipid class has been implicated in age‐related alterations in mitochondrial bioenergetics (Paradies et al., 2019; Semba et al., 2019; Shen et al., 2015). We also observed an accumulation of TGO in the heart, which has been linked to inflammation, endothelial dysfunction, oxidative stress, atherosclerotic plaques, and, ultimately, cardiovascular disease (Figure S2L; Singh & Singh, 2016). TGOs are also closely associated with aging (Johnson & Stolzing, 2019).

Among other metabolic changes, there was an accumulation of nicotinic acid riboside (NaR) in the soleus, cardiac, and gastrocnemius tissues (Figure 6g), indicating potential compensatory mechanisms to maintain NAD^+^ levels, which decline with age in muscle tissues (McReynolds et al., 2021). Linoleic acid, a fatty acid essential for cell membrane integrity and synthesis of inflammation‐related eicosanoids, also increased with age in these three tissues (Figure 6h). In contrast, aminolevulinic acid, which is essential for the production of the heme required for mitochondrial oxygen and energy production (Sawicki et al., 2015), decreased in all three tissues from aged mice (Figure 6i). In summary, our metabolic and lipid profiling analyses revealed significant metabolic alterations in cardiac, soleus, and gastrocnemius muscles with age, some of which may parallel the metabolic alterations resulting from the loss of the MICOS complex.

## 3 DISCUSSION

We demonstrated that either aging or loss of MICOS proteins in skeletal muscle resulted in tissue‐dependent, suboptimal mitochondrial morphology, suggesting a correlation between aging and MICOS protein expression. Previous studies used 3D‐focused ion beam scanning electron microscopy (FIB‐SEM) to characterize the networking of the mitochondria in human (Dahl et al., 2015) and mouse skeletal muscle (Glancy et al., 2015). Quantitative 3D reconstructions used SBF‐SEM to define the morphological differences in the skeletal muscles of humans versus mice and compared patients with primary mitochondrial DNA diseases versus healthy controls (Vincent et al., 2019). However, our current study is the first to use 3D reconstruction to characterize mitochondrial phenotypes in aged skeletal muscles. We used manual contour tracing rather than machine learning techniques to ensure the accuracy of these highly variable mitochondrial phenotypes. Future research is needed to determine whether the human gastrocnemius and soleus muscles have a similar phenotype to murine skeletal muscles. Although the murine and human soleus have similar transcriptomes (Kho et al., 2006), we need further characterization of these muscles as well as oxidative muscle types that may vary in structure and function.

Skeletal muscles depend on mitochondria, comprising ~6% of the cell volume, that change during aging (Garnier et al., 2003). The gastrocnemius muscle has both type I slow‐twitch muscle fibers and type II fast‐twitch muscle fibers; type I fibers are more effective for endurance, whereas type II fibers better support short bursts of muscle activity (Garnier et al., 2003; Lin et al., 2018; Mukund & Subramaniam, 2020). In sarcopenia, the size and frequency of both types of fibers decrease, and type II fibers transition to type I (Romanick et al., 2013). In contrast, muscle atrophy from disuse does not change the fiber number, and there is a shift from type I fibers to type II (Romanick et al., 2013). Thus, age‐related changes in mitochondrial shape may be due to sarcopenia‐dependent alterations in fiber frequency, producing different mitochondrial phenotypes. In our 3D morphologic data, we observed many variable muscle fibers both within a sample and between samples from animals of different ages; however, we could not distinguish the two fiber types, which requires quantitating primary myosin heavy chain proteins after separating them by sodium dodecyl sulfate‐polyacrylamide gel electrophoresis (SDS‐PAGE) (Galpin et al., 2012). In chicken muscle fiber subtypes, there are reportedly large differences in mitochondrial content and morphology; type I does not contain lipid droplets, so the presence of lipid droplets identifies type II fibers (Hosotani et al., 2021; Makida et al., 2022). However, methods for identifying fiber types have not been used with 3D‐reconstructed aged murine skeletal muscles.

Using 3D reconstructions, we found that mitochondria in aged gastrocnemius muscles were smaller in volume, area, and perimeter (Figure 1a–x), and the mitochondria were less interconnected; however, in other tissues, we saw a less significant decrease in volume. Increased fragmentation suggested decreased mitochondrial fusion, which is likely associated with the age‐dependent decrease of OPA‐1, a regulator of mitochondrial fusion. We also saw a decrease in the MCI in aged tissue, suggesting a reduction in mitochondrial networking; however, the mitochondrial shape may not change greatly because of the increased sphericity as they age (Figure 2).

MICOS proteins play key regulatory roles in mitochondrial structure and function (Hu et al., 2020; Li et al., 2016; Wang et al., 2020). By TEM 3D reconstructions, we found that the KD of *Mitofilin*, *Chchd3*, and *Chchd6* in muscles resulted in fragmentation, disrupted cristae, and smaller mitochondria (Figure 4), similar to the loss of *Opa1*, which results in changes in oxidative phosphorylation (Hu et al., 2020; Pereira et al., 2017; Zheng et al., 2019). Overall, mitochondria lacking the MICOS genes had characteristics similar to those of aged mouse skeletal muscle (Figures 1 and 2), and the similarity of the phenotypes suggests an association. Thus, changes in mitochondrial morphology due to aging may be caused by a lack of MICOS protein expression. This is supported by decreased *Chchd3*, *Chchd6*, *Mitofilin*, and *Opa1* transcripts in aged muscle (Figure 3). However, it is possible that despite both skeletal and having lower transcript levels, only skeletal muscle shows reduced MICOS complex and OPA1 protein levels, demonstrating the need to measure age‐dependent protein expression in the future. We found differences in the metabolome and lipidome across different tissue types, which suggests that the MICOS‐complex‐dependent response to aging may differ across cell types with different metabolomes. The tissue‐specific loss of the MICOS complex requires further characterization.

The slight upregulation of *Mitofilin* in *Drosophila* (Figure 3j) suggested that its expression may mitigate the loss of other MICOS complex proteins during aging. In a *Drosophila Mitofilin* KD, there was an inverse correlation between the number of mitochondria and the loss of other MICOS complex components. This suggests different roles for *Mitofilin* in *Drosophila* versus mice and indicates the need for additional studies on *Mitofilin*. Furthermore, the different roles of the MICOS complex proteins need further study. Although there were some common phenotypes associated with all MICOS complex KD mice, *Mitofilin* and *Chchd6* KDs did not reduce proton leak as did the *Chchd3* KD (Figure 5f,p). Although all three of these components are necessary for the stabilization of the MICOS and SAM complexes, and, thus, cristae architecture (An et al., 2012; Darshi et al., 2011; Darshi et al., 2011; Ding et al., 2015; Li et al., 2016), further research is needed to determine whether compensatory increases for specific components of the complex, such as Mitofilin, prevent dysfunctional phenotypes after the loss of certain MICOS complex components.

To better understand the effect of the loss of the MICOS complex, we characterized the metabolome and the lipidome in aged mice and found differences not only between skeletal and cardiac tissue but also between soleus and gastrocnemius (Figure 6). This indicates the importance of understanding tissue‐specific differences in mitochondrial structures we observed. Metabolomics revealed pathways that led to muscular dystrophy, whereas other pathways, such as NaR, rescued the muscles. Cardiac tissue had the greatest change in lipids in aged muscle. This suggests that lipid accumulation may be particularly important in cardiac tissue, consistent with the evidence that lipid metabolism is closely linked with pathology (Chung, 2021). Conversely, in the soleus muscle, there was a decrease in sphingolipids, which may indicate that lipotoxicity is important in the aging soleus muscle. Sphingolipids are also associated with MERC regions prior to and during apoptosis (Mignard et al., 2020). Thus, further studies are needed to characterize the association of mitochondria with lipid droplets or interactions with the endoplasmic reticulum (ER) (Ilacqua et al., 2021) to determine whether the contact sites change during aging to protect against lipotoxicity. In addition, mass spectrometry imaging may reveal changes in the spatial distribution of these metabolites during aging (Hogan et al., 2023). In gastrocnemius tissue, phospholipids decreased (Figure 6), as did linoleic acid, which is important for membrane integrity (Cury‐Boaventura et al., 2004). This suggests altered mitochondrial membrane viscosity in aged tissue, consistent with the age‐dependent increase in viscosity that affects oxidative phosphorylation through modulation of supercomplexes (Dencher et al., 2007). Decreased membrane viscosity, which is associated with age‐related pathologies, including Alzheimer's disease, may be a component of a pathomechanism (Kuter et al., 2016). One potential target of the decreased viscosity may be the MICOS complex, because lipids, including cardiolipin, interact with cristae (Ikon & Ryan, 2017). We only saw a change in cardiolipin in cardiac tissue; however, as this region had the least change in mitochondrial structure, cardiolipin may protect against MICOS‐dependent loss of structure. Thus, the loss of cristae morphology may be associated with an increase in cardiolipin. Therefore, aging may be associated with lipid‐mediated membrane changes that affect the MICOS complex and modulate mitochondrial structure and function.

Age‐related lipidomic and metabolomic changes may be due to age‐dependent alterations in the MICOS complex. Many studies have analyzed the mitochondrial metabolome using mouse skeletal muscles (Bocca et al., 2018; de la Barca et al., 2017, 2019; Garcia‐Cazarin et al., 2011; Garnier et al., 2003; Wortel et al., 2017). We found that loss of *Mitofilin* affected cristae morphology (Figure 4i–k), decreased oxidative phosphorylation (Figure 5a), and may have increased lipid and steroid synthesis, which may be important for the regulation of MERCs and cristae formation. We found an increase in tryptophan and methylhistidine metabolism (Figure 5j) and an increase in taurine metabolism and hypotaurine, a key sulfur‐containing amino acid for fat metabolism. Loss of *Opa1* also changes amino acid and lipid metabolism, similar to the loss of *Mitofilin* (Chao de la Barca et al., 2020; Sarzi et al., 2016; Wasilewski et al., 2012). Steroidogenesis, which makes the membrane less rigid, increased. The loss of *Mitofilin*, *Chchd6*, or *Chchd3* resulted in a decrease in oxidative capacity (Figure 5a–f,k–p). However, increased steroid synthesis may allow the cell to recover bioenergetic functions, as steroids such as estrogen decrease membrane viscosity (Torres et al., 2018). *Mitofilin* is critical for maintaining cristae (Hessenberger et al., 2017; Tarasenko et al., 2017), as cristae junctions and contact sites fall apart with the loss of *Mitofilin* (Kondadi et al., 2020). Cells lacking *Mitofilin* may make steroids to help the membrane reconstitute broken cristae. Just as the loss of *Opa1* results in more MERCs (Rowland & Voeltz, 2012), the loss of *Mitofilin* may increase phospholipids (Figure 5j) as a result of increased smooth MERCs, which are associated with lipid changes (Rieusset, 2018). This is supported by the fact that the biosynthesis of phosphatidylethanolamine and phosphatidylcholine, and the metabolism of arachidonic acid and sphingolipids increased with the loss of *Mitofilin* (Figure 5j). Because these phospholipids aggregate around MERCs and may shuffle into the ER, *Mitofilin* may be a key gene for regulating cristae morphology, with a novel role in regulating mitochondrial metabolism.

*Mitofilin* may be an important target to restore energy production. Loss of *Mitofilin* may lead to ER stress, which, via ATF4, activates amino acid transporters (Han et al., 2013) that then activate mTORC1. ER stress activates mTORC as a result of a decrease in glucose (Wortel et al., 2017). Critically, mTORC1 affects glucose homeostasis (Zhang, Wang, et al., 2021), which may lead to inefficient energy use and result in changes in autophagy. Therefore, if loss of *Mitofilin* increases mTORC1, this may explain why deletion of MICOS in *Drosophila* increases autophagy (Wang et al., 2020). Similarly, loss of *Opa1* increases ER stress (Pereira et al., 2017), and loss of *Mitofilin* may increase amino acid catabolism. If ER stress activates amino acid transporters, branched‐chain amino acids could increase ER stress, resulting in a positive feedback loop that affects the health of the cell, cellular energy, metabolism, and antioxidants. ER stress may also result in the poor performance and fragmentation of mitochondria (Figures 4 and 5), and loss of *Mitofilin* may result in the breakdown of protein pathways that regulate ER stress. Other amino acid pathways, such as homocysteine (Figure 5j), are involved in triglyceride uptake and increased intracellular cholesterol, suggesting that proteins like ATF4 (Wortel et al., 2017) and the MICOS complex (Kozjak‐Pavlovic, 2017; Li et al., 2016) are important during aging. In particular, the MICOS components may prevent mitochondrial fragmentation by blocking ER stress pathways in aging. We showed that genes for several MERC proteins were differentially regulated concomitantly with MICOS complex proteins during aging in *Drosophila* (Tables 1 and 2). Further studies are needed to better understand the role of MICOS in MERC formation and the relationship between smooth MERC and lipid synthesis.

Although *Mitofilin* is the key component of the MICOS complex, the loss of *Chchd3* or *Chchd6* leads to a decrease in and disassembly of all *Mitofilin* subcomplex components in mammals, with abnormal cristae morphology and growth defects (An et al., 2012; Bannwarth et al., 2014; Darshi et al., 2011; Genin et al., 2016, 2018; Park et al., 2010; Piñero‐Martos et al., 2016). Downregulation of *Chchd3* is linked to type 2 diabetes (Eramo et al., 2020). In our metabolomics enrichment dataset (Figure 6t), loss of *Chchd3* or *Chchd6* in mouse myotubes resulted in a preference for alternative energy sources, such as lactate, lactose, and starches. Supplementation of healthy myotubes with galactose leads to a 30% increase in oxidative capacity (i.e., OCR) due to an increase in AMPK phosphorylation and cytochrome c oxidase (COX) activity, thereby forcing cells to become more oxidative to maintain ATP levels (Martin et al., 2021). In our tissues, as oxidative metabolism decreased, anaerobic metabolism and lactate levels increased, forcing cells to produce ATP by anaerobic glycolysis. However, long and high‐level exposure to D‐galactose generates free radicals, which alter MERCs that result in mitochondrial dysfunction and induce aging (Barja, 2014; Kandlur et al., 2020). This is the likely explanation for mitochondrial fragmentation in aged samples and loss of the MICOS complex, which should be investigated further.

In conclusion, we present a quantitative evaluation of mitochondrial morphology in mouse skeletal muscle and cardiac tissue using 3D reconstructions, with TEM studies of cell lines to characterize other factors such as cristae architecture. We found structural changes, including changes in gross 3D mitochondrial structure, which produced functional differences upon loss of MICOS proteins. Similar changes in mitochondrial morphology were observed in aged muscles and after the loss of MICOS proteins in mouse skeletal muscle, and we found that MICOS mRNA transcripts decreased with age. We also found that the metabolome and lipidome were heavily altered in aged muscles, suggesting a role for the MICOS complex in membrane integrity during aging. In vivo *Drosophila* models demonstrated the importance of understanding the tissue‐specific responses to aging, the roles of individual components in the MICOS complex, and potential MICOS complex‐MERC pathway interactions that may regulate mitochondrial structure and function. Despite a link between aging and the loss of *Opa1* (Tezze et al., 2017; Varanita et al., 2015), little is known about the role of the MICOS complex in aging. A reduction in MICOS proteins could result in changes in mitochondrial architecture and loss of integrity; thus, we need therapies to restore MICOS proteins and *Opa1* lost during aging to mitigate the deleterious effects of mitochondrial dysfunction. Although knockouts can determine the role of specific proteins in mitochondrial dynamics, few studies have attempted to restore MICOS proteins in mitochondria (Liu et al., 2020; Zheng et al., 2019). Our results established a relationship between the MICOS complex and aging; thus, further studies using 3D reconstruction could elucidate the link between sarcopenia, the MICOS complex, and the role of mitochondria in aging and certain diseases.

## 4 EXPERIMENTAL PROCEDURES

### 4.1 Animal care and maintenance

All procedures for the care of mice were in accordance with humane and ethical protocols approved by the University of Iowa Animal Care and Use Committee (IACUC), or the University of Washington IACUC, following the National Institute of Health (NIH) Guide for the Care and Use of Laboratory Animals, as described previously (Pereira et al., 2017). Therefore, all studies are performed in accordance with the ethical standards established in the 1964 Declaration of Helsinki and its later amendments. All experiments used WT male C57Bl/6J mice housed at 22°C on a 12‐h light, 12‐h dark cycle with free access to water and standard chow. Mice were anesthetized with 5% isoflurane/95% oxygen and followed by cervical dislocation.

### 4.2 RNA extraction and RT‐qPCR

Total RNA was extracted from tissue using TRIzol reagent (Invitrogen; cat #15596026), purified with the RNeasy kit (Qiagen Inc; cat #74004), and quantitated by the absorbance at 260 and 280 nm using a NanoDrop 1000 (NanoDrop products) spectrophotometer. Total RNA (~1 μg) was reverse transcribed using a High‐Capacity cDNA Reverse Transcription Kit (Applied Biosciences; cat #4368814), followed by real‐time quantitative PCR (qPCR) reactions using SYBR Green (Life Technologies; cat #S7563) (Boudina et al., 2007). Triplicate technical replicates for qPCR (~50 ng) in a 384‐well plate were placed into ABI Prism 7900HT instrument (Applied Biosystems) programmed as follows: 1 cycle at 95°C for 10 min; 40 cycles of 95°C for 15 s; 59°C for 15 s, 72°C for 30 s, and 78°C for 10 s; 1 cycle of 95°C for 15 s; 1 cycle of 60°C for 15 s; and 1 cycle of 95°C for 15 s. Data were normalized to glyceraldehyde‐3‐phosphate dehydrogenase (*Gapdh*), and results are shown as fold changes. qPCR primers were designed using Primer Blast or were previously published sequences (Pereira et al., 2017), as shown in Table 3.

**TABLE 3:** qPCR primers used.

Gene	Primers	
*Opa1*	Forward	5′‐ACCAGGAGACTGTGTCAA‐3′
	Reverse	5′‐TCTTCAAATAAACGCAGAGGTG‐3′
*Chchd3*	Forward	5′‐GAAAAGAATCCAGGCCCTTCCACGCGC‐3′
	Reverse	5′‐CAGTGCCTAGCACTTGGCACAACCAGGAA‐3′
*Chchd6*	Forward	5′‐CTCAGCATGGACCTGGTAGGCACTGGGC‐3′
	Reverse	5′‐GCCTCAATTCCCACATGGAGAAAGTGGC‐3′
*Mitofilin*	Forward	5′‐CCTCCGGCAGTGTTCACCTAGTAACCCCTT‐3′
	Reverse	5′‐TCGCCCGTCGACCTTCAGCACTGAAAACCTAT‐3′

### 4.3 Experimentally evolved Drosophila populations, RNA extraction, RT‐qPCR

Groups of experimentally derived *Drosophila melanogaster* were selected using different generation cycles for hundreds of generations to produce populations with different patterns of aging and longevity. In the control populations, termed CO_1–5_, genetically diverse populations (census size, ~2000 per replicate) were maintained on a 28‐day generation cycle. From these CO populations, a new population was maintained on a 9‐day cycle, ACO_1–5,_ for hundreds of generations, as described in Chippindale et al. (1997). This resulted in an ACO population that evolved to reproduce earlier, develop more rapidly and die much earlier than the CO population (Burke et al., 2016). The accelerated aging in the ACO flies is associated with differences in genetics (Graves et al., 2017), patterns of gene expression (Barter et al., 2019), and the metabolome (Phillips et al., 2022). We refer to the ACO populations as “aged flies” and the CO population as “control flies.”

We used RT‐qPCR to compare gene expression for the genes as shown in Table 2 in cardiac tissue from 21‐day‐old flies from the ACO and CO populations. We chose Day 21 based on demographic data (Burke et al., 2016) and whole‐body transcriptomic data (Barter et al., 2019) showing large differences between the two populations at that age. For each group, we collected heart tissue using the following protocols. On Day 21 from eggs, female fruit flies from each of the 10 ACO and CO populations were anesthetized using Fly Nap (Carolina), a triethylamine‐based anesthetic, for about 1 min or until no movement was detected. Flies were dissected in oxygenated artificial hemolymph to expose the cardiac tubes, and the abdomens were opened to remove guts/intestines, fat, and ovaries. Although it was not possible to fully remove fat and pericardial cells from the cardiac tube without damaging it, excess fat and pericardial cells were carefully suctioned away from the cardiac tube in the exposed hearts (Vogler & Ocorr, 2009). Three biological replicates were collected for each ACO and CO population, and each biological replicate comprised 18–20 pooled adult hearts. Total RNA was extracted using Qiazol (Qiagen) and miRNeasy Mini Kit (Qiagen; cat #217004) with DNase digestion (Qiagen RNase‐Free DNase set; cat #79254). RNA was reverse transcribed to cDNA using a QuantiTect Reverse Transcription Kit (Qiagen). Three replicates from ACO and CO populations were selected after filtering for DNA concentration (1 ng/μL) and purity (260/280 ratio > 1.8). Duplicate samples in wells that contained 7.5 μL of 2× iTaq SYBR Green Supermix (Bio‐Rad Laboratories), 3 μL of ddH_2_O, 0.75 μL of each primer, and 3 μL of template cDNA were run on a CFX96 Touch thermal cycler (Bio‐Rad Laboratories). The PCR program was 1 cycle at 95°C for 2 min, 40 cycles at 95°C for 30 s, and 60°C for 30 s; however, genes *dMic60*, *Bip*, and *Ire1* were run at an annealing temperature of 62°C. The melt curves were analyzed for quality control (QC). Data are expressed as ∆∆*C*_t_ and normalized to the *Drosophila* gene rp49/RpL32. Primers designed using Primer Blast are shown in Table 4.

**TABLE 4:** *Drosophila* qPCR primer sequences.

Gene	Primers
*ATF‐4*	F: 5′‐TCGCAAAAGTTGGTTAAACG‐3′ R: 5′‐TCCGTAGGATTCAACTGCTG‐3′
*Drp1*	F: 5′‐TCCACAATCTTCTCGTGCAG‐3′ R: 5′‐CATTCACGAGGAGATGCAGC‐3′
*Marf*	F: 5′‐GTATGTCCATGAGACGACCA‐3′ R: 5′‐CTTGTACACATAGCTTTCGA‐3′
*Opa1*	F: 5′‐GACTCTGACCGGGAATACGA‐3′ R: 5′‐CTAGAACCATGCGTTGCAGA‐3′
*MINOS1 (Mic10)*	F: 5′‐GTCCTTTTAACGTTGTTTTGGCA‐3′ R: 5′‐AGCGATGCCCATTCCAAATC‐3′
*QIL1 (Mic13)*	F: 5′‐AGACTCTGACCAGACGGACA‐3′ R: 5′‐AGGGCAGCATGTGGATGAAA‐3′
*CHCHD3/6 (mic19)*	F: 5′‐GCTAGAGGAACTTCAAAGATGG‐3′ R: 5′‐GGGATAGGAGGATACTTTCGG‐3′
*Dmic60 (mitofilin)*	F: 5′‐TCCAAAACATAAATACTCTGGGAAG‐3′ R: 5′‐ACAAAGCTTGCCAATTTCAGC‐3′
*APOO (mic26/27)*	F: 5′‐CGGTCTGGCTGGTTTCATCT‐3′ R: 5′‐GGCACTACGGGAACATCCTC‐3′
*VDAC/Porin*	F: 5′‐ATCTGAAGACTAAGACCTCGTCG‐3′ R: 5′‐AGACCTTTCCAGACTCCTGGT‐3′
*IP3R/Itpr*	F: 5′‐ATGGGCGACAATATAATTGGCTC‐3′ R: 5′‐GTGCTCAAGAAACCGCAAACG‐3′
*FGF21/bln*	F: 5′‐TGTCGCCCGCTGACAATAAT‐3′ R: 5′‐TTGCTGATGGGCGTGTTACT‐3′
*Ire1*	F: 5′‐GAACGCGAGTGCGAAGAAAA‐3′ R: 5′‐CTGATGCAATAAGCCCGCTG‐3′
*Bip(grp78)/Hsc 70‐3*	F: 5′‐CGCATCGAAATTGAATCCTT‐3′ R: 5′‐TTCAGGGTGGAACGGAATAG‐3′
*ATF‐6*	F: 5′‐TGAGCCTAATTCGTCTCCCAC‐3′ R: 5′‐TAGACCGCCTCTTCGTTAGAA‐3′
*RP49*	F: 5′‐AGGCCCAAGATCGTGAAGAA‐3′ R: 5′‐TCGATACCCTTGGGCTTGC‐3′
*GRP75/Hsc70‐5*	F: 5′‐CGCGTACCCAAGTTTCTGC‐3′ R: 5′‐CGGAACATGCTAGAAGCTCC‐3′

### 4.4 Drosophila strains and genetics

For genetic crosses, flies were grown on yeast corn medium (Katti et al., 2017, 2022) at 25°C. The Mef2‐Gal4 strain served as a control within their respective genetic backgrounds. Mef2‐Gal4 was crossed to the genetic background w1118 to generate the UAS‐RNAi knockdown lines per previous protocols (Ranganayakulu et al., 1996). Mef2 encodes the transcription factor myocyte enhancer factor‐2, which regulates muscle development. Gal4 is a transcriptional activator from yeast commonly used to drive gene expression in Drosophila when cloned upstream of a promoter region. In this study, Mef2‐Gal4 refers to a transgenic fly line expressing Gal4 under the control of the Mef2 promoter (Ranganayakulu et al., 1996). Male and female flies were analyzed collectively, as there were no discernible sex differences in mitochondrial morphology in WT muscles. Mef2‐Gal4 (III) was utilized for muscle‐specific knockdown of MICOS genes. The mitochondrial network was visualized using UAS‐mito‐GFP, located on the second chromosome (BS# 8442). For muscle‐specific knockdown of MICOS genes, UAS‐RNAi transgenic RNAi project (TRiP) lines were used for UAS‐*Chchd3* RNAi (BS#51157), UAS‐*Mitofilin* RNAi (BS# 63994), UAS‐QIL1 RNAi (BS# 44634), UAS‐Drp1 RNAi (BS# 51483), UAS‐Marf RNAi (BS# 55189), and UAS‐Opa1 RNAi (BS#32358). All stocks were acquired from the Bloomington *Drosophila* stock center and denoted by the Bloomington Stock Number (BS#). All chromosome designations and gene symbols are described in FlyBase (http://flybase.org).

### 4.5 Mitochondrial staining

Adult *Drosophila* thoraces, aged 2–3 days, were dissected in 4% paraformaldehyde (PF; Sigma) using fine scissors and processed as described by Katti et al. (2022) (Miller et al., 2019), and isolated indirect flight muscles were fixed in 4% PF for 1.5 h using a rotator, followed by three 15‐min washes in PBSTx (phosphate buffered saline [PBS] + 0.3% Triton X‐100). Mitochondria were visualized using either the fluorescent mitochondrial stain Mito Tracker Red (M22425; Thermofisher) or green fluorescent protein (GFP) via *Drosophila* Mef2‐Gal4 (Bloomington Drosophila Stock Center, stock #27390) driven UAS‐mito‐GFP. Actin was stained by incubating muscles in 2.5 μg/mL of Phalloidin in PBS (Sigma, 1 mg/mL stock of Phalloidin TRITC) at 25°C for 40 min. Stained tissues were mounted on a glass slide using Prolong Glass Antifade Mountant with NucBlue Stain (P36985; Thermofisher). Images were captured using a Zeiss 780 confocal microscope.

### 4.6 Mitochondrial measurements

Mitochondrial measurements have been described previously (Haas, 2019; Lam et al., 2021), and images were analyzed using the NIH ImageJ software (https://imagej.net). Individual mitochondria were outlined on 2D light microscopic images using the freehand tool provided by the software, and their area and aspect ratio (the ratio of the major axis to the minor axis) were calculated using ImageJ. For each data set, three animals were analyzed, and these analyses were part of three independent experiments conducted to gather quantifiable data. The number of mitochondria was counted across every three‐sarcomere segment.

### 4.7 Isolation of satellite cells

Satellite cell differentiation was performed as described previously (Lam et al., 2021; Pereira et al., 2017). Gastrocnemius muscles were dissected from 8 to 10 week‐old WT mice and washed twice with 1× PBS supplemented with 1% penicillin–streptomycin (Gibco) and Fungizone (300 mL/100 mL). Dulbecco's Modified Eagle *Medium*/Nutrient Mixture F‐12 (DMEM‐F12) with collagenase II (2 mg/mL), 1% penicillin–streptomycin, and Fungizone (300 mL/100 mL) was added to the muscle which was agitated for 90 min at 37°C. For the second wash, collagenase II was changed to 0.5 mg/mL, and the muscle was agitated for 30 min at 37°C. The tissue was cut, passed through a 70‐mm cell strainer, and after centrifugation, satellite cells were plated on BD Matrigel‐coated dishes. To differentiate cells into myoblasts, a mixture of DMEM‐F12, 20% fetal bovine serum (FBS) (Atlanta Bio selected), 40 ng/mL basic fibroblast growth factor (bFGF, R and D Systems, 233‐FB/CF), 1× non‐essential amino acids, 0.14 mM β‐mercaptoethanol, and 1× penicillin–streptomycin, and Fungizone was used. Myoblasts were maintained with 10 ng/mL bFGF, and when cells reached 80% confluence, myoblasts were differentiated in DMEM‐F12, 2% FBS, 1× insulin–transferrin–selenium medium. Cells were cultured at 37°C with 5% CO_2_ in DMEM (GIBCO) supplemented with 10% FBS and 1% penicillin–streptomycin.

### 4.8 CRISPR‐Cas9 knockouts

After 3 days, myotubes were incubated with CRISPR/Cas9 mixtures to produce the following knockouts—control CRISPR/Cas9 (sc‐418922), *Chchd6* (Mic25) CRISPR (sc‐425817), *Chchd3* (Mic19) CRISPR (sc‐425804), and *Mitofilin* (Mic60) CRISPR (sc‐429376) (Santa Cruz Biotechnology), with the use of a guide RNA (Table 5). We incubated 2.5% relevant CRISPR, 2.5% RNAiMax (ThermoFisher Scientific; cat #13778075), and 95% Opti‐MEM (Gibco; cat #31985070) in a tube for 20 min. Cells were washed twice with PBS after removal of the medium; then, 800 μL of OPT‐MEM (Gibco; cat #31985062) and 200 μL of the CRISPR mixture were added to each well and were run in triplicate. Cells were incubated for 4 h at 37°C, 1.0 mL of DMEM medium was added, and cells were incubated overnight. The myotubes were then washed with PBS, and the medium was replaced. Experiments were performed between 3 and 7 days after knockout for a total of 6 days of differentiation.

**TABLE 5:** Guide RNA and plasmids used.

Gene name	Type of plasmid	CAS number
*Mitofilin*	CRISPR/Cas9 KO (mouse)	sc‐429376
*Chchd6*	CRISPR/Cas9 KO (m)	sc‐425817
*Chchd3*	CRISPR/Cas9 KO (m)	sc‐425804
*Control*	CRISPR/Cas9 KO (m)	sc‐418922

### 4.9 Serial block‐face scanning electron microscope (SBF‐SEM) processing of mouse muscle fibers

SBF‐SEM preparation was performed as described previously (Garza‐Lopez et al., 2022; Hinton et al., 2023; Neikirk et al., 2021). Male mice were anesthetized with 5% isoflurane, the hair and skin were removed, and the hindlimbs were incubated in 2% glutaraldehyde with 100 mM phosphate buffer for 30 min. Gastrocnemius muscles were dissected, cut into 1 mm^3^ cubes, and incubated in 2.5% glutaraldehyde, 1% paraformaldehyde, and 120 mM sodium cacodylate solution for 1 h. Tissues were washed three times with 100 mM cacodylate buffer at room temperature before immersion in 3% potassium ferrocyanide and 2% osmium tetroxide for 1 h at 4°C, then treated with 0.1% thiocarbohydrazide, 2% filtered osmium tetroxide for 30 min, and left overnight in 1% uranyl acetate at 4°C. Between each step, three deionized water washes were performed. The following day, samples were immersed in 0.6% lead aspartate solution for 30 min at 60°C and dehydrated in graded concentrations of acetone. Dehydration was for 5 min each in 20%, 50%, 70%, 90%, 95%, and 100% acetone. Tissues were impregnated in Epoxy Taab 812 hard resin, then embedded in fresh resin, and polymerized at 60°C for 36–48 h. Once polymerization was complete, blocks were sectioned for TEM to identify areas of interest, trimmed to 0.5 mm × 0.5 mm, and glued to aluminum pins. The pins were run on an FEI/Thermo Scientific Volumescope 2 SEM, a state‐of‐the‐art SBF imaging system, yielding 300–400 10 μm by 10 μm ultrathin (90 nm) serial sections, as per previous techniques (Garza‐Lopez et al., 2022). All sections were collected onto formvar‐coated slot grids (Pella), stained, and imaged as described previously (Garza‐Lopez et al., 2022; Hinton et al., 2023; Neikirk et al., 2021).

### 4.10 Quantification of TEM micrographs and parameters using ImageJ

Quantification of TEM images was performed as described previously using NIH ImageJ software (Hinton et al., 2023; Lam et al., 2021). Cells were divided into four quadrants, and two quadrants were selected randomly for complete analysis. Individuals blinded to the experimental design measured a minimum of 10 cells using three analyses to obtain accurate and reproducible values. When there was variability, we assigned 30 cells per individual to reduce the variability.

### 4.11 Measurement of OCR using seahorse

We used the Seahorse XF24 extracellular flux (XF) bioanalyzer (Agilent Technologies/Seahorse Bioscience) to measure cellular respiration. Cells were plated at a density of 2 × 10^4^ per well and differentiated. After 3 days of differentiation, *Opa‐1*, *CHCHD3*, *CHCHD6*, or *Mitofilin* genes were knocked out as described above. Three days after knockout, the medium was changed to XF‐DMEM (Agilent‐103680), and cells were kept in a non‐CO_2_ incubator for 60 min. The basal OCR was measured in XF‐DMEM. Oxygen consumption was measured after the addition of oligomycin (1 μg/mL), carbonyl cyanide 4‐(trifluoromethoxy)phenylhydrazone (FCCP; 1 μM), rotenone (1 μM), and antimycin A (10 μM) (Pereira et al., 2017; Wende et al., 2015). Cells were then switched to glucose‐free XF‐DMEM and kept in a non‐CO_2_ incubator for 60 min for the glycolysis stress test. Seahorse experimental data used triplicate Seahorse plates. Three independent experiments were performed with four to six replicates for each time and each condition, and representative data from the replicates are shown.

### 4.12 Segmentation and quantification of 3D SBF‐SEM images using Amira

The mitochondria analyzed were the IMF mitochondria located between myofibrils that are arranged in pairs at the z‐band of each sarcomere, with 2D elongated tubular shapes (Vendelin et al., 2005). For each ROI across the two age groups, we analyzed 300 slices at 50 μm intervals in transverse intervals. For 3D reconstruction, SBF‐SEM images were segmented manually using Amira software (Thermo Scientific) as described previously (Garza‐Lopez et al., 2022; Hinton et al., 2023). All serial sections (300–400 slices) were loaded onto Amira, and structural features were traced manually on sequential slices of micrograph blocks. Structures in mice were collected from 30 to 50 serial sections that were then stacked, aligned, and visualized using Amira to make videos and quantify volumetric structures. An average of 500 total mitochondria across four ROIs from three mice was collected for quantification. For the 3D reconstruction of myotubes, approximately 20 mitochondria from a minimum of 10 cells were collected. Quantification of SBF‐SEM images was performed as described previously (Garza‐Lopez et al., 2022) using the Amira software.

### 4.13 Western blotting

Tissues from adult (3 months) and aged (2 years) mice were lysed with RIPA lysis buffer (1% NP40, 150 mM NaCl, 25 mM Tris base, 0.5% sodium deoxycholate, 0.1% SDS, 1% phosphatase inhibitor cocktails #2 (Sigma P5726‐1ML) and #3 (Sigma P0044‐1ML), and one cOmplete protease inhibitor tablet (Sigma 04693159001)). Protein was quantified using a BCA Assay (Thermo Scientific VLBL00GD2), and equal amounts of protein were run on 4%–20% Tris‐glycine gels (Invitrogen WXP42012BOX). Protein was transferred to a nitrocellulose membrane (Li‐Cor 926‐31092) that was incubated with primary antibodies overnight at 4°C: MTOC1 (Invitrogen PA5‐26688), phospho S406 ATGL (Abcam ab135093), DRP1 (CST 8570S), pDRP1 (CST 6319S), OPA1 (BD Biosciences 612306), Mic60/mitofilin (Abcam ab110329), SLC25A46 (Abcam ab237760), SAM50 (Proteintech 20824‐1‐AP), or tubulin (Novus NB100‐690). Secondary antibodies were diluted to 1:10,000 and incubated with the membrane at room temperature for 1 h: donkey anti‐mouse IgG (H + L) (Invitrogen A32789) and donkey anti‐rabbit IgG (H + L) (Invitrogen A32802). Blots were imaged with the Li‐Cor Odyssey CLx infrared imaging system.

### 4.14 Gas chromatography–mass spectrometry (GC–MS) for MICOS knockouts

Samples were extracted for metabolites and prepared as described previously (Phillips et al., 2022; Yoon et al., 2022).

Samples were extracted in −80°C 2:2:1 methanol/acetonitrile/water that contained a mixture of nine internal standards (d_4_‐citric acid, ^13^C_5_‐glutamine, ^13^C_5_‐glutamic acid, ^13^C_6_‐lysine, ^13^C_5_‐methionine, ^13^C_3_‐serine, d_4_‐succinic acid, ^13^C_11_‐tryptophan, d_8_‐valine; Cambridge Isotope Laboratories), each at a concentration of 1 μg/mL and at a ratio of 18:1 (extraction solvent:sample). Cells were lyophilized overnight before extraction and homogenized with a ceramic bead mill homogenizer after the addition of extraction buffer. Samples were incubated for 1 h at −20°C and centrifuged at maximum speed for 10 min. All supernatants were transferred to fresh tubes, and pooled QC samples were prepared by adding an equal volume of each sample to a new 1.5 mL microcentrifuge tube. A speed vac was used to evaporate the pooled QCs, samples, and processing blanks, which were made by adding extraction solvent to microcentrifuge tubes. Derivatives of the dried products were obtained using methoxamine hydrochloride and *N*,*O*‐bis(trimethylsilyl)trifluoroacetamide (TMS). Products were rehydrated in 30 μL of 11.4 mg/mL molybdenum carbide in anhydrous pyridine (VWR), vortexed for 10 min, and incubated at 60°C for 1 h. Then, 20 μL of TMS was added to the samples, which were vortexed for 1 min and heated for an hour at 60°C. Samples of 1 μL were analyzed by GC–MS using a Thermo Trace 1300 GC with a TraceGold TG‐5SilMS column for GC chromatographic separation. The GC inlet temperature was 250°C, with the oven temperature set at a gradient with 3 min at 80°C, increasing by 20°C/min to the final 280°C temperature for the last 8 min. The settings for the GC machine were 20:1 split ratio; split flow, 24 μL/min; purge flow, 5 mL/min; carrier mode, constant flow; and carrier flow rate, 1.2 mL/min. The column was washed three times with pyridine between each injection sample. Metabolites were detected using the Thermo ISQ single quadrupole mass spectrometer, with data acquired from 3.90 to 21.00 min in the EI mode (70 eV) by single‐ion monitoring. We used TraceFinder 4.1, with standard verified peaks and retention times, to profile the metabolites and to compare metabolite peaks in each sample against an in‐house library of standards. For these standards, we analyzed retention times and fragment ions for each, with fragment ions for both the target peak and two confirming ions. For the samples, we identified metabolites that matched both retention times and the three fragment ions. TraceFinder was also used for GC–MS peak integration to obtain peak areas for each metabolite. After this analysis, we used previously described protocols (Li et al., 2017) to correct for drift over time by using QC samples run at the beginning and end of the sequence. The data were then normalized to an internal standard to control for extraction, derivatization, and/or loading effects.

### 4.15 Liquid chromatography–mass spectrometry (LC–MS) for MICOS KO

Myotubes were dried, rehydrated in 40 μL acetonitrile:water (1:1), and vortexed. For LC–MS, 2 μL of the sample was used with a Thermo Q Exactive hybrid quadrupole Orbitrap mass spectrometer with a Vanquish Flex UHPLC system and a Millipore SeQuant ZIC‐pHILIC column (length area = 2.1 × 150 mm, 5 μm particle size) with a ZIC‐pHILIC guard column (length area = 20 × 2.1 mm). The mobile phase comprised solvent A (20 mM ammonium carbonate [(NH_4_)_2_CO_3_] and 0.1% ammonium hydroxide [NH_4_OH]) and solvent B (acetonitrile). The mobile phase gradient started at 80% solvent B, decreased to 20% solvent B over 20 min, returned to 80% solvent B in 0.5 min, and was held at 80% for 7 min (Cantor et al., 2017). From there, the mass spectrometer was operated in the full‐scan, polarity‐switching mode for 1–20 min, spray voltage set to 3.0 kV, capillary heated at 275°C, and HESI probe heated at 350°C. The sheath gas flow, auxiliary gas flow, and sweep gas flow were 40 units, 15 units, and 1 unit, respectively. We examined an *m*/*z* range of 70–1000, the resolution was set at 70,000, the automatic gain control (AGC) target at 1 × 10^6^, and the maximum injection time was set to 200 ms (Li et al., 2017). TraceFinder 4.1 software was used for analysis, and metabolites were identified based on an in‐house library. Drift was corrected for as described above (Li et al., 2017). Data were normalized, and further visualization and analysis were performed on MetaboAnalyst 5.0 (Chong et al., 2018).

### 4.16 Analyzing metabolomic data for MICOS KO

Metabolomic analysis was performed as described previously (Phillips et al., 2022) using the web service MetaboAnalyst 5.0 (https://www.metaboanalyst.ca/MetaboAnalyst/ModuleView.xhtml, last accessed on 8 February 2022) that combines machine learning methods and statistics to group data using PCA, heat mapping, metabolite set enrichment analysis, and statistical analysis. One‐way ANOVA and Fisher's least significant difference multiple comparison tests were also used. PCA uses score plots to provide an overview of variance for the principal components. Heatmaps separate hierarchical clusters leading to progressively larger clusters. Clusters are based on similarity using Euclidean distance and Ward's linkage to minimize the clustering needed. Metabolite set enrichment analysis, which determines whether a set of functionally related metabolites is altered, identifies consistent changes across many metabolites with similar roles. Overrepresentation analysis determines whether a group of compounds is overrepresented compared to chance and whether a group of metabolites has similar changes. In this analysis, the fold enrichment was calculated by dividing the observed hits by the expected metabolites. The expected number of hits was calculated by MetaboAnalyst 5.0. GraphPad Prism software was used for statistical analysis with data expressed as mean ± standard deviation, and one‐tailed *p*‐values ≤ 0.01 were considered significant.

### 4.17 Metabolomics on aged samples

Metabolomic analysis was performed as described previously (Adusumilli & Mallick, 2017; Lu et al., 2018; Wang et al., 2019). Frozen tissues from aged mice were weighed, ground in liquid nitrogen in a cryo‐mill (Retsch) at 25 Hz for 45 s, extracted in 40:40:20 acetonitrile:methanol:water +0.5% formic acid +15% NH_4_HCO_3_ (Lu et al., 2018) in 40 μL of solvent per 1 mg of tissue, vortexed for 15 s, and incubated on dry ice for 10 min. Samples were centrifuged at 16,000 × g for 30 min, transferred to new microcentrifuge tubes, and then centrifuged again at 16,000 × g for 25 min to remove residual debris.

Extracts were analyzed within 24 h by LC–MS, based on hydrophilic interaction chromatography (HILIC) coupled to the Orbitrap Exploris 240 mass spectrometer (Thermo Scientific) (Wang et al., 2019). The LC separation was performed on an XBridge BEH Amide column (2.1 × 150 mm, 3.5 μm particle size; Waters). Solvent A was 95%:5% H_2_O:acetonitrile with 20 mM ammonium acetate and 20 mM ammonium hydroxide, and solvent B was 90%:10% acetonitrile:H_2_O with 20 mM ammonium acetate and 20 mM ammonium hydroxide. The gradient was 0 min, 90% B; 2 min, 90% B; 3 min, 75% B; 5 min, 75% B; 6 min, 75% B; 7 min, 75% B; 8 min, 70% B; 9 min, 70% B; 10 min, 50% B; 12 min, 50% B; 13 min, 25% B; 14 min, 25% B; 16 min, 0% B; 18 min, 0% B; 20 min, 0% B; 21 min, 90% B; and 25 min, 90% B. The parameters for the LC analysis were a flow rate of 150 mL/min, column temperature of 25°C, injection volume of 5 μL, and autosampler temperature of 5°C. For the detection of metabolites, the mass spectrometer was operated in both negative and positive ion modes. The parameters for the MS analysis were a resolution of 180,000 at *m/z* 200, AGC target at 3 × 10^6^, maximum injection time of 30 ms, and a *m/z* scan range of 70–1000. Raw LC/MS data were converted to mzXML format using the command line “msconvert” utility (Adusumilli & Mallick, 2017). Data were analyzed via the EL‐MAVEN software version 12.

### 4.18 Lipidomics of aged samples

#### 4.18.1 Tissue homogenization and extraction of lipids

Tissues were ground as described in the section above. The homogenate was mixed with 1 mL of extraction buffer containing isopropyl alcohol (IPA)/H_2_O/ethyl acetate (30:10:60, v/v/v) and Avanti Lipidomix Internal Standard (diluted 1:1000) (Avanti Polar Lipids, Inc.). Samples were vortexed and homogenized twice in a VWR Bead Mill at 6000 × g for 30 s. The samples were then sonicated for 5 min and centrifuged at 15,000 × g for 5 min at 4°C. The upper phase was transferred to a new tube and kept at 4°C. The tissue pellet was again extracted using 1 mL of IPA/H_2_O/ethyl acetate extraction buffer, vortexed, homogenized, sonicated, and centrifuged as described above. The supernatants from both extractions were combined, and the organic phase was dried under liquid nitrogen gas.

#### 4.18.2 Sample reconstitution for lipids

The dried samples were reconstituted in 300 μL of solvent A (IPA/acetonitrile/H_2_O, 45:35:20, v/v/v). Samples were vortexed briefly, sonicated for 7 min, and centrifuged at 15,000 × g for 10 min at 4°C. The supernatants were transferred to clean tubes and centrifuged again for 5 min at 15,000 × g at 4°C to remove any remaining particulates. For LC–MS lipidomic analysis, 60 μL of the sample extracts was transferred to mass spectrometry vials.

#### 4.18.3 LC–MS analysis for lipids

Sample analysis was performed within 36 h after extraction using a Vanquish UHPLC system coupled with an Orbitrap Exploris 240™ mass spectrometer equipped with an H‐ESI™ ion source (all Thermo Fisher Scientific) and a Waters CSH C18 column (1.0 × 150 mm × 1.7 μm particle size). Solvent A consisted of acetonitrile:H_2_O (60:40; v/v) with 10 mM ammonium formate and 0.1% formic acid, and solvent B contained IPA: acetonitrile (95:5; v/v) with 10 mM ammonium formate and 0.1% formic acid. The mobile phase flow rate was 0.11 mL/min, and the column temperature was 65°C. The gradient for solvent B was as follows: 0 min, 15% (B); 0–2 min, 30% (B); 2–2.5 min, 48% (B); 2.5–11 min, 82% (B); 11–11.01 min, 99% (B); 11.01–12.95 min, 99% (B); 12.95–13 min, 15% (B); and 13–15 min, 15% (B). Ion source spray voltages were set at 4000 and 3000 V in positive and negative modes, respectively. Full‐scan mass spectrometry was conducted with a scan range from 200 to 1000 *m/z*, and AcquireX mode was utilized with a stepped collision energy of 30% with a 5% spread for fragment ion MS/MS scan.

#### 4.18.4 Lipidomics analysis

After normalization, all data were analyzed in R using the *lipidr* package (Mohamed et al., 2020). All code to analyze data and generate figures can be found at https://github.com/mphillips67/Lipidomic‐Analysis‐Young‐and‐Aged‐Mouse‐Tissue. Data sets for each tissue type were analyzed independently. Data were log‐transformed and further processed before analysis. For lipids with multiple readings across replicates, only the sets of readings with the highest values were used. In a few instances (2–3), for each tissue type, all samples had identical measurements. As this was likely due to technical errors, these samples were not used. Lastly, to be compatible with *lipidr*, lipid names had to be modified to fit a standard “CLS xx:x/yy:y” naming scheme where CLS refers to the abbreviated lipid class and xx:x and yy:y refer to the first and second chains (*note*: a code to generate a conversion key is available through the GitHub link above).

The lipid composition of young and old samples was compared after processing using the “de_analysis” function from *lipidr* with default settings. Here, *lipidr* uses moderated *t*‐tests to identify significant differences in lipids between sample types within a tissue type. Significantly different lipids were those with adjusted *p*‐values < 0.05 (*note*: *p*‐values were adjusted to correct for multiple comparisons using a false discovery rate procedure) and log fold changes greater than 1 or less than −1. These results were used to perform a lipid set enrichment analysis using the “lsea” function in which entries were ranked by fold change; only classes with at least four associated lipids were considered, and 100,000 permutations were run. Here, the method *lipdr* used is based on the commonly used gene set enrichment analysis approach previously outlined (Subramanian et al., 2005). Briefly, lipid class and chain length categories were determined from annotations extracted from lipid names in the data set, and lipids were ranked by fold change. A permutation algorithm was used to calculate enrichment scores and *p*‐values for each lipid set. Sets with adjusted *p*‐values < 0.05 were defined as significantly enriched. Lastly, heatmaps were generated for significantly enriched lipid classes using the “plot_heatmap”.

### 4.19 Data analysis

All SBF‐SEM and TEM data are presented as the mean of at least three independent experiments with similar outcomes. Results are presented as mean ± standard error of the mean (SEM) with individual data points shown. Data from only two groups were analyzed using an unpaired *t*‐test. For nanotunnel quantification, a Mann–Whitney (unpaired, nonparametric) *t*‐test was performed between the two groups. If more than two groups were compared, one‐way ANOVA was performed, and significance was assessed using Fisher's protected least significant difference test. GraphPad Prism software package was used for *t*‐tests and ANOVA analyses. For all statistical analyses, *p* < 0.05 indicated a significant difference. Higher degrees of statistical significance (**, ***, ****) were defined as *p* < 0.01, *p* < 0.001, and *p* < 0.0001, respectively.

## AUTHOR CONTRIBUTIONS

Z.V., E.G., L.V., J.S., H.K.B., S.A.M., M.A.P., M.R.M., A.H.J., J.A.G., and D.D. conceived and designed the research; A.G.M., A.C., L.V., Z.V., T.A.C, B.C.M., J.L., H.K.B., B.R, C.E., D.D., A.C.M., B.C.J., P.P, M.R.M, A.H.J., and J.A.G. performed experiments; J.D., K.N, J.S., E.G., Z.V., J.L., B.R., T.A.C., A.K.R., A.M.Q., V.E., E.G., D.D., A.C.M., B.C.J., P.P, M.R.M., J.A.G., and A.H.J. analyzed data; B.T, K.N, J.S., E.G., Z.V., S.A.M., A.M.Q., V.E., H.K.B., A.C., A.G.M., J.D., M.A.P., M.R.M., D.D., J.A.G., and A.H.J. interpreted the results of experiments and prepared figures; K.N, E.G., Z.V., J.S., S.A.M., L.V., A.G.M., M.A.P., A.K.R., B.C.M., B.T., C.E., A.C., H.K.B., M.R.M., D.D., J.A.G., and A.H.J. drafted, edited, and revised the manuscript; M.R.M., A.H.J., D.D., and J.A.G. approved the final version of manuscript.

## CONFLICT OF INTEREST STATEMENT

All authors declare that they have no conflict of interest.

## FUNDING INFORMATION

This project was funded by the National Institute of Health (NIH) NIDDK T‐32, number DK007563 entitled Multidisciplinary Training in Molecular Endocrinology to Z.V. and A.C.; Integrated Training in Engineering and Diabetes, Grant Number T32 DK101003; Burroughs Wellcome Fund Postdoctoral Enrichment Program #1022355 to D.S.; The UNCF/Bristol‐Myers Squibb (UNCF/BMS)‐E.E. Just Postgraduate Fellowship in Life sciences Fellowship and Burroughs Wellcome Fund/PDEP #1022376 to H.K.B.; NSF MCB #2011577I to S.A.M.; NIH K01AG062757 to M.T.S.; NSF EES2112556, NSF EES1817282, NSF MCB1955975, and CZI Science Diversity Leadership grant number 2022‐253614 from the Chan Zuckerberg Initiative DAF, an advised fund of Silicon Valley Community Foundation to S.D.; The UNCF/Bristol‐Myers Squibb E.E. Just Faculty Fund, Career Award at the Scientific Interface (CASI Award) from Burroughs Welcome Fund (BWF) ID # 1021868.01, BWF Ad‐hoc Award, NIH Small Research Pilot Subaward to 5R25HL106365‐12 from the National Institutes of Health PRIDE Program, DK020593, Vanderbilt Diabetes and Research Training Center for DRTC Alzheimer's Disease Pilot & Feasibility Program. CZI Science Diversity Leadership grant number 2022‐253529 from the Chan Zuckerberg Initiative DAF, an advised fund of Silicon Valley Community Foundation to A.H.J.; and National Institutes of Health grant HD090061 and the Department of Veterans Affairs Office of Research Award I01 BX005352 to J.G. Howard Hughes Medical Institute Hanna H. Gray Fellows Program Faculty Phase (Grant# GT15655 awarded to M.R.M); and Burroughs Wellcome Fund PDEP Transition to Faculty (Grant# 1022604 awarded to M.R.M). Additional support was provided by the Vanderbilt Institute for Clinical and Translational Research program supported by the National Center for Research Resources, Grant UL1 RR024975‐01, and the National Center for Advancing Translational Sciences, Grant 2 UL1 TR000445‐06 and the Cell Imaging Shared Resource. The contents are solely the responsibility of the authors and do not necessarily represent the official view of the NIH. The funders had no role in study design, data collection, and analysis, decision to publish, or preparation of the manuscript. NIH Grants R01HL147818, R03HL155041, and R01HL144941 (A. Kirabo).

## CONSENT FOR PUBLICATION

All authors have agreed to the final version of this manuscript.

## Supporting information


Figures S1–S2

Tables S1–S4

## Data Availability

The data that support the findings of this study are available from the corresponding author upon reasonable request. Permissions: The authors of this article previously showed Figure 1 preliminary findings as proceedings in Microscopy and Microanalysis, Volume 29, Issue Supplement_1, on 1 August 2023. All rights have been obtained and are availabe from the corresponding author upon request.
