# Supplementary material for: 3D reconstruction of murine mitochondria reveals changes in structure during aging linked to the MICOS complex
Source: Aging Cell. 2023 Nov 13;22(12):e14009. doi: 10.1111/acel.14009 (PMC10726809; doi:10.1111/acel.14009)
Supplement: Supplementary file 1 — Figures S1–S2 [file ACEL-22-e14009-s002.zip › Legends.docx]

**Supplementary:**

**Figure S1: Distribution of mitochondria among mice samples.**

Distribution in each mouse of mitochondrial volume in (**A**) gastrocnemius, (**B**) soleus, and (**C**) cardiac tissue from 3-month-old and 2-year-old mice to represent individual heterogeneity. Similar data are shown for (**D–F**) mitochondrial area, (**G–I**) mitochondrial perimeter, (**J–L**) mitochondrial complexity index, and (**M–O**) mitochondrial sphericity in gastrocnemius, soleus, and cardiac tissue from 3-month-old and 2-year-old mice.

**Figure S2: Metabolomics and lipidomic analysis revealed metabolic dysregulation and disruptions in lipid classes and chain lengths in aged gastrocnemius, soleus, and cardiac muscles.** (**A**) Volcano plot of metabolites that were differentially represented in aged compared to young gastrocnemius, (**B**) soleus, and (**C**) cardiac samples. For volcano plots, the x-axis represents the median, and the y-axis represents the adjusted FDR. (**D**) Enrichment analysis for metabolites enriched in aged gastrocnemius, (**E**) soleus, and (**F**) cardiac muscles. (**G**) PCA plot for young and aged gastrocnemius, (**H**) soleus, and (**I**) cardiac muscles. (**J**) Lipid class fold-change ratios between young and old tissues in gastrocnemius, (**K**) soleus, and (**L**) cardiac muscles. Significantly different lipid classes represented in the figures are those with adjusted p-values < 0.05 and log fold changes greater than 1 or less than −1. (**M**) Lipid chain length fold-change ratios between young and old tissues in gastrocnemius, (**N**) soleus, and (**O**) cardiac muscles. Significantly different lipid chain lengths represented in the figures are those with adjusted p-values < 0.05 and log fold changes greater than 1 or less than −1.

**Supplementary Tables:**

Table S1. Lipid classes present in our dataset and associated abbreviations.

Table S2. Results for set enrichment analysis, lipid class, and chain length, based on lipidomic differences between young and old gastrocnemius tissue.

Table S3. Results for set enrichment analysis, lipid class, and chain length, based on lipidomic differences between young and old soleus tissue.

Table S4. Results for set enrichment analysis, lipid class, and chain length, based on lipidomic differences between young and old cardiac tissue.
