# Supplementary figures and images for: 3D reconstruction of murine mitochondria reveals changes in structure during aging linked to the MICOS complex
Source: Aging Cell. 2023 Nov 13;22(12):e14009. doi: 10.1111/acel.14009 (PMC10726809; doi:10.1111/acel.14009)

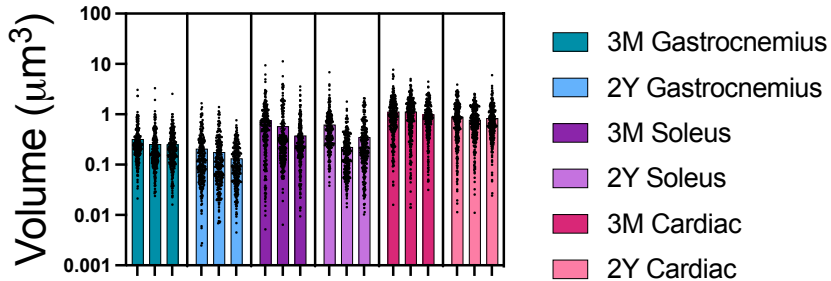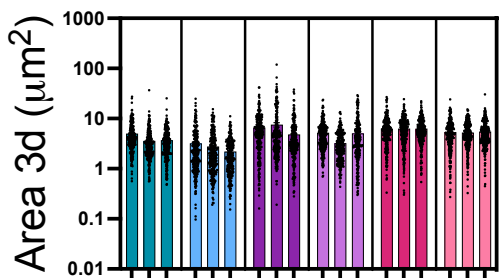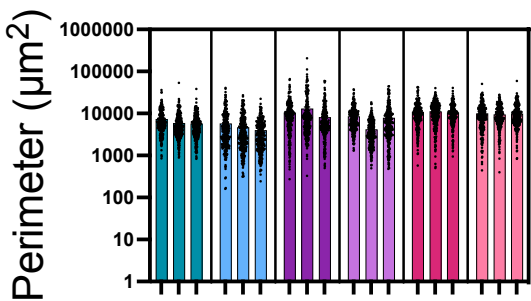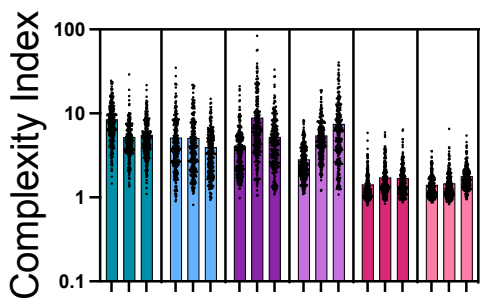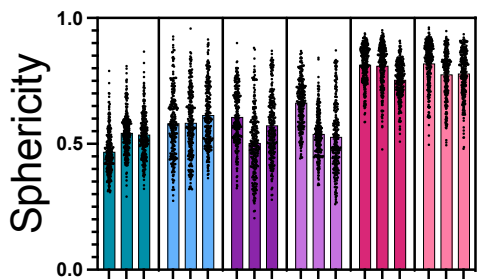

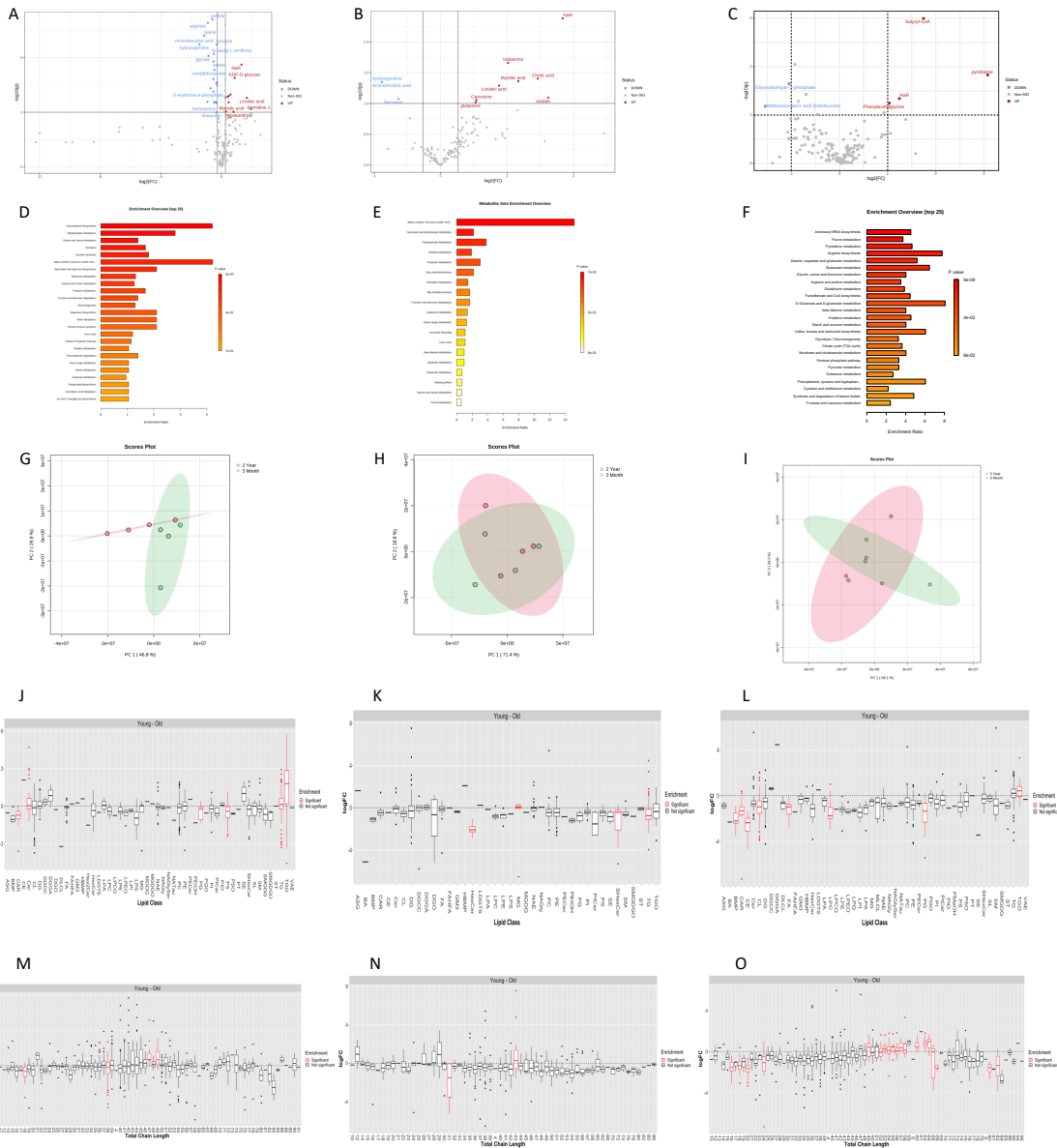

Supplement: Supplementary file 1 — Figures S1–S2 [file ACEL-22-e14009-s002.zip › acel14009-sup-0001-FiguresS1-S2.pdf]
